# Phylogeny of the Viral Hemorrhagic Septicemia Virus in European Aquaculture

**DOI:** 10.1371/journal.pone.0164475

**Published:** 2016-10-19

**Authors:** Michael Cieslak, Susie S. Mikkelsen, Helle F. Skall, Marine Baud, Nicolas Diserens, Marc Y. Engelsma, Olga L. M. Haenen, Shirin Mousakhani, Valentina Panzarin, Thomas Wahli, Niels J. Olesen, Heike Schütze

**Affiliations:** 1 Institute of Infectology (IMED) of the Friedrich-Loeffler-Institut (FLI), Federal Research Institute for Animal Health, Insel Riems, Germany; 2 Section for Virology, National Veterinary Institute, DTU, Frederiksberg, Denmark; 3 Fish Health, Department of Animal Science, Aarhus University, Tjele, Denmark; 4 Ploufragan-Plouzané Laboratory (ANSES), Viral Fish Pathology Unit, Université Européenne de Bretagne, Technopôle Brest-Iroise, Plouzané, France; 5 Centre for Fish and Wildlife Health (FIWI), Vetsuisse-Faculty, University of Bern, Bern, Switzerland; 6 Central Veterinary Institute of Wageningen UR, NRL for Fish and Shellfish Diseases, Lelystad, the Netherlands; 7 Istituto Zooprofilattico Sperimentaledelle Venezie, OIE Reference Laboratory for Viral Encephalopathy and Retinopathy, Legnaro, Italy; Universidade Federal do Rio de Janeiro, BRAZIL

## Abstract

One of the most valuable aquaculture fish in Europe is the rainbow trout, *Oncorhynchus mykiss*, but the profitability of trout production is threatened by a highly lethal infectious disease, viral hemorrhagic septicemia (VHS), caused by the VHS virus (VHSV). For the past few decades, the subgenogroup *Ia* of VHSV has been the main cause of VHS outbreaks in European freshwater-farmed rainbow trout. Little is currently known, however, about the phylogenetic radiation of this *Ia* lineage into subordinate *Ia* clades and their subsequent geographical spread routes. We investigated this topic using the largest *Ia*-isolate dataset ever compiled, comprising 651 complete *G* gene sequences: 209 GenBank *Ia* isolates and 442 *Ia* isolates from this study. The sequences come from 11 European countries and cover the period 1971–2015. Based on this dataset, we documented the extensive spread of the *Ia* population and the strong mixing of *Ia* isolates, assumed to be the result of the Europe-wide trout trade. For example, the *Ia* lineage underwent a radiation into nine *Ia* clades, most of which are difficult to allocate to a specific geographic distribution. Furthermore, we found indications for two rapid, large-scale population growth events, and identified three polytomies among the *Ia* clades, both of which possibly indicate a rapid radiation. However, only about 4% of *Ia* haplotypes (out of 398) occur in more than one European country. This apparently conflicting finding regarding the Europe-wide spread and mixing of *Ia* isolates can be explained by the high mutation rate of VHSV. Accordingly, the mean period of occurrence of a single *Ia* haplotype was less than a full year, and we found a substitution rate of up to 7.813 × 10^−4^ nucleotides per site per year. Finally, we documented significant differences between Germany and Denmark regarding their VHS epidemiology, apparently due to those countries’ individual handling of VHS.

## Introduction

The rainbow trout, *Oncorhynchus mykiss* (Walbaum, 1792), is one of the dominant fish species in European aquaculture in terms of produced weight and value. In 2014, 272,938 metric tons of rainbow trout (from inland waters, brackish waters, and marine waters) [1] worth about 1,000,000 USD were produced in Europe (approximately 4 USD per kilo) [2]. European rainbow trout production is threatened, however, by viral hemorrhagic septicemia (VHS), which annually leads to substantial economic losses in the trout farming industry. These losses can be a direct consequence of fish mortality or an indirect consequence of disease control measures. In view of its large economic impact, VHS is categorized as a notifiable disease by the OIE (World Organization for Animal Health) [3] and the European Union (Council Directive 2006/88/EC) [4]. The etiological agent of VHS is the VHS virus (VHSV), an enveloped single-stranded, negative-sense RNA virus that belongs to the genus *Novirhabdovirus* in the family *Rhabdoviridae* [5]. The genome of this virus contains approximately 11,200 nucleotides and encodes six proteins in the following arrangement: 3′-N-P-M-G-NV-L-5′ (a non-structural protein (NV) and five structural proteins: nucleoprotein (N), phosphoprotein (P), matrix protein (M), glycoprotein (G), and RNA polymerase (L)) [6, 7].

During acute VHS infection, fish do exhibit external disease signs such as a darkening of the skin, bulging eyes, anemia, bleeding on the skin, gills, eyes, and internal organs, and a bloated abdomen [8]. Mortality can be as high as 100% in fry, but ranges from 5% to 90% in older rainbow trout [8, 9]. VHSV is transmitted through contact with the urine or reproductive fluids of other infected fish and also through virus-contaminated water or objects [8]. VHS is commonly a cool- or cold-water disease that is most prevalent at temperatures of 9–12°C [8].

The first records of a rainbow trout exhibiting symptoms similar to those of the current VHS disease are from German trout farms in the early 1930s [10]. Soon after, the disease was reported from several other European countries [9]. However, the viral agent in this disease was not successfully isolated until the early 1960s [11].

Until the mid-1970s, VHS was considered to be a disease specific to trout in Europe, but in the decades that followed, VHSV was also isolated from a variety of farmed and wild fish species in Europe, North America, Japan, and Korea; VHSV has now been isolated from more than 80 wild and farmed fish species [8]. The chronology of VHSV isolation did not occur in parallel with a simultaneous spreading event of VHSV, however, it resulted mainly from an increase in monitoring efforts, which initially focused mostly on European rainbow trout farms [9].

Europe harbors its own endemic VHSV genogroups in comparison to North America and Asia. Whereas genogroups *I* (marine and freshwater), *II* (marine), and *III* (marine) are endemic to Europe, genogroup *IV* (marine and freshwater) has almost exclusively been isolated from North American and Asian fish. Currently, there is substantial evidence that these genogroups had already split before the 20^th^ century [12, 13] and therefore long before the virus was discovered in North America (1988) [14] and Asia (1996) [15].

Genogroup *I* is further divided into six subgenogroups: *I*_*(unclassified)*_ (Denmark, freshwater), *Ia* (predominantly continental Europe), *Ib* (Northern Europe, marine), *Ic* (continental Europe), *Id* (Scandinavia–Baltic Sea and freshwater), and *Ie* (Black Sea region); and genogroup *IV* is divided into three subgenogroups: *IVa* (North American Pacific Coast), *IVb* (Great Lakes), and *IVc* (North American Atlantic Coast) [12, 13, 16, 17]. This phylogenetic classification has largely been derived from sequences of the *G* and *N* genes.

In Europe, VHS has a major adverse effect on the production of freshwater-farmed rainbow trout, but disastrous VHS outbreaks have also been recorded in a few cases of farmed turbot (*Scophthalmus maximus*) and brown trout (*Salmo trutta*) [18–21]. Since the collection of VHSV isolates from European freshwater rainbow trout farms began in 1962, most VHS outbreaks have been found to be caused by subgenogroup *Ia* isolates [22].

He and colleagues (2014) suggested that the ancestral *Ia* lineage arose from a pathogenic virus of freshwater-farmed rainbow trout during the 1950s in France [13]. Little is still known, however, about the *Ia* lineage radiation into *Ia* clades and their respective spread routes. Currently, there is a hypothesis that the *Ia* lineage can be divided into two sublineages (*Ia*-1 and *Ia*-2) [22]. This hypothesis is based on the observation that most of the isolates from the *Ia*-1 group were sampled from Danish farms, whereas isolates from the *Ia*-2 group were largely from other European countries.

Given that in RNA viruses (such as the VHSV), the accumulation of mutational changes and biogeographical processes (e.g., an event of spread, vicariance, extinction, or rapid population growth) take place at similar temporal scales due to the high substitution rate [23], it is assumed that the phylogeny and phylogeographic pattern of VHSV will provide important insights into the epidemiology of VHS. Accordingly, in this study, we sought to shed new light on the following topics: (1) the phylogenetic radiation of the *Ia* lineage into subordinate *Ia* clades; (2) the country-specific distribution and individual spread route of each *Ia* clade; and finally, (3) the question if there are indications for a hard polytomy, or a sudden demographic expansion, which would point to a rapid radiation. Due to the particularly high spatiotemporal density of Danish and German *Ia* isolates in our dataset from approximately the last 20 years, we particularly focused our phylogenetic analysis on those two countries during this period.

Our study was carried out within the scope of a multidisciplinary trans-European research project, MOLTRAQ (molecular tracing of viral pathogens in aquaculture), by using the largest dataset of *Ia* isolates ever compiled. We believe this work will provide an enhanced epidemiological understanding of European VHS disease, and our goal is to contribute the epidemiological knowledge that is a basic prerequisite for developing and implementing efficient VHSV prevention and eradication measures in the future.

## Materials and Methods

### Ethics statement

Ethical approval was not required for this study. VHSV samples were obtained from European health services and regional laboratories and were isolated from fish on the basis of the COUNCIL DIRECTIVE 2006/88/EC of the European Union on animal health requirements for aquaculture animals and products thereof, and on the prevention and control of certain diseases in aquatic animals [4].

### Collection of VHSV isolates

We sampled 452 VHSV isolates: 2 from Austria, 1 from the Czech Republic, 152 from Denmark, 11 from France, 222 from Germany, 21 from Italy, 1 from Kattegat (Scandinavian sea area), 3 from the Netherlands, 37 from Switzerland, and 2 from Turkey. These samples covered the period from 1977–2015 (S1 Table). The viruses were sampled and isolated according to the standardized methods described in 2001/183/EC [24]. The date of collection, site of collection, and host species of each isolate are summarized in the S1 Table. Viral isolates were propagated at 15°C in the cell lines RTG-2 (CCLV Rie 686) and EPC (CCLV Rie 173). Moreover, 282 *G* gene sequences of VHSV isolates from GenBank (NCBI: National Center for Biotechnology Information) were also incorporated into our sample collection. For inclusion, the sequence of the complete *G* gene, the date of collection, and the geographic site of collection all had to be recorded. Finally, our complete VHSV dataset comprises 734 isolates from Austria, Denmark, France, Georgia, Germany, Poland, Slovenia, Switzerland, Turkey, the United Kingdom, waters of the Atlantic and Northern Coast of Central Europe, waters of Northern Europe, and the North American Pacific Coast, collected from 1971–2009 (S1 Table).

### RNA extraction

Total RNA was extracted from the infected cell culture after two freeze-thaw cycles using the RNeasy mini kit from Qiagen. Then 1.2 mL of the sample was centrifuged at 14,000 rpm for 60 min at 12°C and the resulting pellet was dissolved in 600 μL of RLT buffer. The remaining extraction steps were performed according to the manufacturer’s instructions. Lastly, the RNA was eluted in 30μL of RNase-free water.

### RT-PCR and *G* gene sequencing

Primers for RT-PCR and sequencing were designed based on the published sequence of VHSV in the GenBank database under the accession number Y18263 (S2 Table). The sequence of the complete *G* gene was amplified using the primers V2782for and V4664rev (S2 Table). The predicted 1883-bp RT-PCR product (VHSV genome: nucleotides 2782–4664) encompasses the 1524-bp-long *G* gene, which is localized between nucleotides 2959 and 4482 of the VHSV genome (accession number: Y18263) [25]. RT-PCR was performed using the One Step RT-PCR Kit (Qiagen). The 25-μL reaction mixture consisted of the one-step RT-PCR buffer (Qiagen, including 12.5 mM MgCl_2_), 40 mM deoxynucleosidetriphosphate (dNTP) mix (10mM of each dNTP), 10 pmol each of the forward and reverse primers, 1.0 μL of the enzyme mix (Qiagen), 20U of RNasin (Promega), and 10–100 ng of total extracted RNA. The following PCR cycling conditions were used: 45 min at 45°C for reverse transcription; 15 min at 95°C for initial activation of DNA polymerase and for inactivation of reverse transcriptase and denaturation of the cDNA template; and then 35 cycles of 94°C for 30 s (denaturation), 57°C for 30 s (annealing), and 68°C for 10 min (extension). The reactions were conducted in an automated thermal cycler (MastercyclerGradient, Eppendorf).

RT-PCR products were analyzed on 0.7% agarose gels in TAE buffer, and products of the predicted size (approximately 1.9 kb) were eluted by using a QIAquick gel-extraction kit (Qiagen) according to the manufacturer’s instructions. Next, 50 ng of the eluted RT-PCR product was sequenced directly using specific primers as listed in S2 Table; the sequence of both DNA strands was determined by cycle sequencing using a Big Dye Terminator version 1.1 Cycle Sequencing kit (Applied Biosystems) according to the manufacturer’s instructions. The sequencing product was purified using either Sigma Spin Post-Reaction purification columns (Sigma Aldrich) or Nucleo Spin Columns (Machery Nagel). After denaturation with Hi-Di Formamide, samples were analyzed on an automatic sequencer (ABI 377, Applied Biosystems), and nucleotide sequences were evaluated using the sequencer’s Scanner software version 1.0 (ABI) and Geneious software version 7.1.7 (Biomatters Ltd.) [26]. The full-length *G* gene sequence was deposited in GenBank and the Fish Pathogens Database [27].

### Determination of the haplotype

The haplotype of a VHSV isolate was determined based on the substitution differences within the complete *G* gene sequence. Specimens that featured a variation of two possible nucleotides at a single position of the *G* gene sequence were split into two haplotypes and phylogenetically handled as though they were two isolates. However, if the specimen varied at more than one nucleotide position of the *G* gene sequence, they were excluded from the dataset. Multiple sequence alignment was performed using Geneious Pro version 7.1.7 (Biomatters Ltd.) [26]. The number of haplotypes was calculated using DnaSP version 5.10.01 [28].

### Phylogenetic subdivision of the *Ia* subgenogroup (*Ia* lineage) into *Ia* clades

Following the principle the lower the bootstrap support value, the less reliable the grouping, we divided the haplotypes of the *Ia* subgenogroup into subordinate *Ia* clades on the basis of a Maximum Likelihood (ML) bootstrap support value of ≥75%. The bifurcating ML tree, with each node splitting into exactly two descendant branches, was constructed by the computer program MEGA version 5.2 [29]. The best-fit nucleotide substitution model was selected using the Bayesian Information Criterion score with Find Best DNA Model in MEGA version 5.2 [29]. As a result, the general time reversible (GTR) model with gamma rate heterogeneity and invariant sites was chosen, and 250 bootstrap replicates were generated to assess the reliability of the clades obtained in the tree. In addition, we further divided those *Ia* clades with a more complex structure in the MJ network into subsets. This extra division was to better illustrate the phylotemporal arrangement of their haplotypes, or to group those haplotypes that form a star-like pattern for a separate analysis.

### Identification of a polytomy in the phylogeny to indicate a potential hard polytomy

A polytomy means that a node splits into more than two nodes. However, in case of a phylogenetic tree (in our case the bifurcating ML tree), its bifurcation algorithm does not permit the correct phylogenetic resolution of a polytomy. Normally, there are two possible causes for a polytomy. The first possible cause is taxon sampling bias [30, 31] due to a lack of sufficient data or inappropriate analysis of characters. The second possible cause is a hard polytomy [30, 31]. This means that a lineage splits into more than two descendant lineages, also referred to as multifurcation. Such polytomy nodes can occur, for example, when isolates (with a specific haplotype) were simultaneously separated into more than two different environments (e.g., geographic regions). After this, different local scenarios of selection and genetic drift result in the development of individual sublineages (clades) with a common time of origin. Therefore, a hard polytomy can be interpreted as a rapid radiation event. It is widely recognized that polytomy relationships are relatively common in intraspecific gene phylogenies [32].

Although an extensive dataset of *Ia* isolates is available for this study, a sampling bias cannot generally be excluded. Therefore, detected polytomies can be interpreted as a potential indicator for a hard polytomy rather than evidence for such. To discover nodes with a tendency toward polytomy we adopted the following procedure. First, we created a phylogenetic Median Joining (MJ) network using the computer program NETWORK version 4.6.1.2 (http://www.fluxus-engineering.com) [33]. This method is well suited to represent the polytomy structure of a node. The program’s default setting of Epsilon (0) was chosen and the transition/transversion bias (R) was based on a maximum likelihood estimate obtained using MEGA version 5.2 [29]. Due to the lack of a robustness measure for the MJ network in the NETWORK program, we sought to enhance the reliability of the MJ network topology by examining: (1) if the haplotypes of each ML *Ia* clade are also grouped in the MJ network; and (2) if they have a phylotemporal structure. In case of the latter, the expectation would be that older isolates (earlier collection dates) would cluster closer to the ancestral node of the lineage than isolates with more recent collection dates. Secondly, we created a NeighborNet (NN) network [34] using the program SplitsTree version 4.13.1 [35]. This method is also well suited to represent the polytomy structure of a node, as it can present alternative parallel branches (splits) in the phylogenetic topology and even allows bootstrapping. We generated 1,000 bootstrap replicates to assess the reliability of the splits obtained in the splits graph. Finally, for each polytomy node (noticed in the MJ and NN networks), we examined whether such a node was associated with a bifurcation conflict in the ML tree. For this purpose, we used a quick principle: the number of internal nodes is less than the number of tips minus 1 [36]. In other words, each polytomy node leads to at least one additional node (depending on the number of descendant lineages, for example: 1, in the case of a trifurcating node; 2, in the case of a quadfurcating node; and so forth) with a low bootstrap support value, which is only present in the ML tree, and not in the MJ nor NN networks.

### The mean period of occurrence in years of a *Ia* haplotype or clade

The mean period of occurrence, in years, of a single *Ia* haplotype, or an *Ia* clade, was calculated using the following criteria: 0, occurrence within one calendar year; 1, occurrence within two calendar years; and so forth.

### Phylogeographic analysis and nucleotide diversity

The phylogeographic pattern of the *Ia* population was illustrated by the MJ network constructed by NETWORK version 4.6.1.2 [33]. Nucleotide diversity (π) was calculated using DnaSP version 5.10.01 [28].

### Indication of rapid population growth

A rapid increase in the size of an *Ia* clade population (a sudden demographic expansion due to increased population growth) was indicated using the following methods. (1) Visual inspection for a major star-like pattern in the MJ phylogenetic network constructed by NETWORK version 4.6.1.2 [33]. (2) A coalescent Bayesian skyline analysis [37, 38]. The Bayesian skyline plot was constructed using the program BEAST version 1.8.2 [39] and was visualized with the program Tracer version 1.5 (from BEAST). A further output of that method was the meanRate (substitution rate) of the sequence used for this calculation. The BEAST input gene files were generated using BEAUti version 1.8.2. Our analyses used the random starting tree for the Markov chain Monte Carlo (MCMC) search, selecting a general time GTR model along with an uncorrelated lognormal relaxed molecular clock. We ran 90 million cycles sampling every 9,000 cycles. Outputs were assessed in TRACER version 1.5 to ensure that values reached stationarity. (3) Statistical tests, including the raggedness index (r), Tajima’s D [40], and Fu’s Fs [41], using DnaSP version 5.10.01 [28]. (4) A mismatch distribution analysis [42, 43], also using DnaSP version 5.10.01 [28]. However, the above mentioned methods may not separate the effects of rapid population growth from a recent selective sweep [44]. Therefore, their results can only be used as an indicator for such a demographic event.

## Results

We successfully sequenced 452 VHSV isolates, and they fell into the previously published phylogenetic classification of genogroups on the basis of their haplotypes (1 *I*_*(unclassified)*_ isolate, 442 *Ia* isolates, 1 *Ib* isolate, 6 *Ic* isolates, and 2 *Ie* isolate) (S1 Table). The GenBank and Fishpathogens database accession numbers of the additional sequences obtained for this study are also listed in S1 Table. In total, the dataset (GenBank samples and samples sequenced for this study) consists of 734 VHSV isolates (4 *I*_*(unclassified)*_ isolates, 651*Ia* isolates, 27 *Ib* isolates, 11 *Ic* isolates, 32 *Id* isolates, 6 *Ie* isolates, and 1 each of isolates *II*, *III*, and *IV*). Genogroup *Ia* isolates can be subdivided into 398 haplotypes (S1 Table). Approximately 95% of these 651 *Ia* isolates were obtained from *Oncorhynchus mykiss*, 3% from *Salmo trutta*, 1% from *Esox* lucius, and 1% from 6 further fish species (isolates from an unknown host species are excluded) (S1 Table).

**Fig 1:**
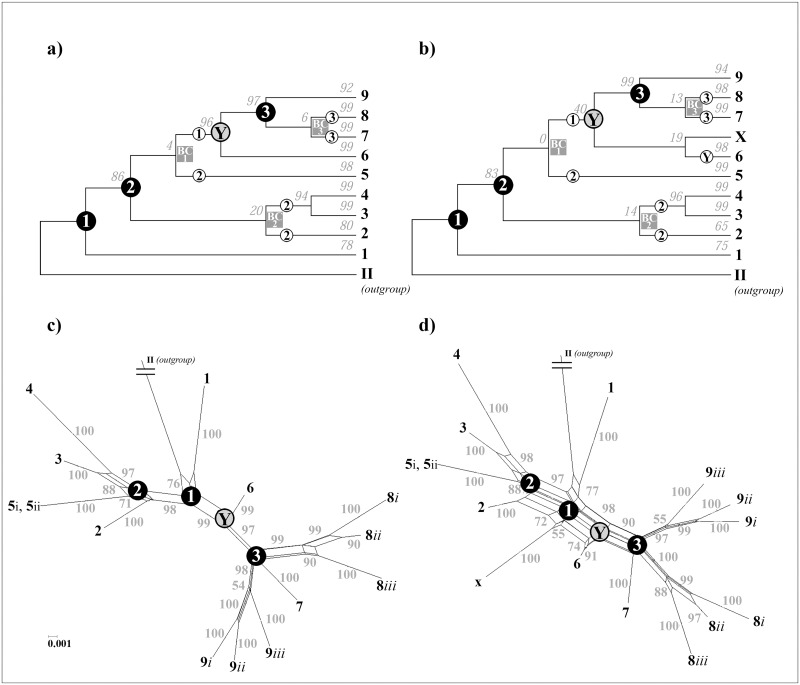
Maximum Likelihood trees and NeighborNet networks of the *Ia* subgenogroup. In diagrams a) and b) the phylogenetic relationship between the *Ia* clades (clades *1*–*9*), based on the complete *G* gene sequence, is illustrated as a bifurcating maximum likelihood tree, whereas in diagrams c) and d) it is shown as a NeighborNet network. Diagram a) is based on 650 *Ia* isolates (S1 Table), diagram b) on 651 *Ia* isolates (the same isolates as in a) plus the isolate U288000, designated as X). Diagrams c) is based on isolates with the GenBank accession-number AY546571 (9*i*), LN877188 (9*ii*), EU708732 (9*iii*), EU708755 (8*iii*), EU708748 (8*ii*), LN877010 (8*i*), LN876935 (7), AJ233396 (6), LN876803 (5), EU708742 (4), FRG2192 (3), LN876782 (2), and AY546617 (1), diagram d) is based on the same isolates as in c) plus the isolate X. These isolates each represent the *Ia* clade isolate with the oldest collection date. Isolate AY546576 (VHSV genogroup II) is the outgroup in each diagram. The phylogenetic trees are pictured as cladograms. Numbers above branches represent the bootstrap support values obtained from 250 replicates. In case of the networks, the formation of parallelograms indicates possible alternative split events, and the small gray numbers are bootstrap values for each branch (shown only for values>50%). Black circles marked “1–3” represent nodes that correspond in the networks with a polytomy. Gray squares marked “BC1–3” represent nodes that occur only in the trees due to a bifurcation conflict. In the tree diagrams, the bootstrap support value of node Y was increased from 40% to 96% when isolate X was excluded. Small white circles marked “1–3” on a branch indicate the respective connection of this branch to the node 1–3 in the networks.

**Fig 2:**
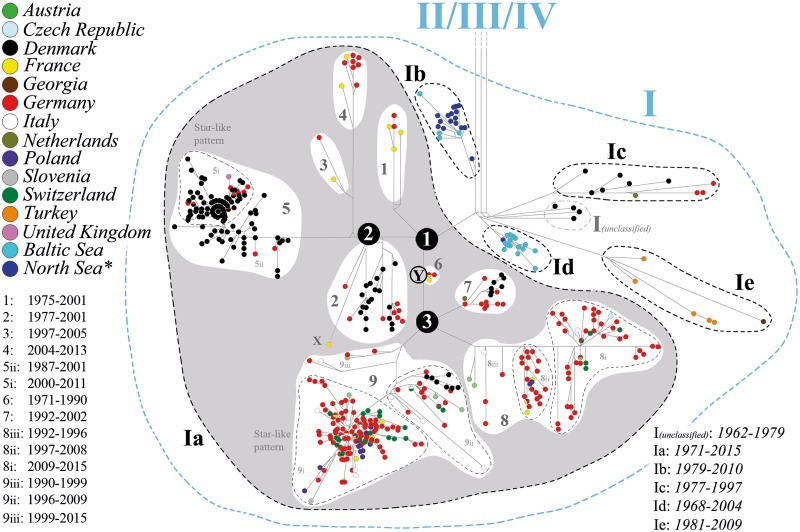
Median-joining network based on the complete *G* gene sequence from 734 VHSV isolates (transition/transversion bias = 5). The country or region of origin is indicated using a color code. The North Sea (labeled by an asterisk) includes also the Barents Sea, English Channel, Kattegat, Norwegian Sea, Rogaland, and Skagerrak. The years represent the range of collection dates for isolates of every European subgenogroup *I* (*I(unclassified)*, and *Ia–e*) and *Ia* clades (clade *1–9*). The clades *5*, *8*, and *9* are additionally subdivided into the subsets *5i–iii*, *8i–iii*, and *9i–iii*. The black circles with the number 1 and 3 represent clear polytomy nodes (node 1 and 3), whereas the black circle with the number 2 represents a intermediate stage between a polytomy and bifurcating node (node 2). The node labeled by the gray circle Y may represents the bifurcating node between the *Ia* clades, or it may represent the more or less direct ancestor of the polytomy node 3.

We divided the *Ia* haplotypes into nine clades (*Ia* clades *1*–*9*). Their ML bootstrap support values ranged from 78% and 99% (mean value = 94%; standard deviation = 8.62%) (Fig 1a). This division was identical to the *Ia*-haplotype clade structure in the MJ phylogenetic network (Fig 2). A striking exception was isolate U28800 (hereafter referred to as isolate X) that was the only taxon in the *Ia* dataset with varying clade-membership when comparing the ML, MJ, and NN *Ia* phylogeny. In the ML tree, isolate X clustered with clade *6* (Fig 1b), but with a very low bootstrap support value of 19%. In contrast, in the MJ network, the lineage of X originated from the common ancestor of clade *2* (Fig 2), whereas in the case of the NN network, it originated close to the ancestral node of the *Ia* lineage (node 1), which was approximately between the origins of the lineage of clade *2* and the lineage of clade *6* (Fig 1d). However, when isolate X is excluded from the dataset, the bootstrap support value of node Y increased from 40% to 96% (Fig 1a and 1b). In addition, the number of splits was significantly lower in the NN network of Fig 1c in comparison to Fig 1d.

The *Ia* phylogeny in the MJ network was largely characterized by a phylotemporal structure: older *Ia* clades (those that included isolates with earlier collection dates) clustered closer to the phylogenetic origin of the *Ia* lineage (node 1) than did clades with more recent isolates. For example, clades *1*, *2*, and *6* include haplotypes from the oldest collected *Ia* isolates (Fig 2 and S1 Table). Moreover, we further divided the *Ia* clades *5*, *8*, and *9* into subsets (clade *5* into subset *5i*–*ii*, *8* into subset *8i*–*iii*, and *9* into subset *9i*–*iii*) to better illustrate the phylotemporal arrangement of their haplotypes, and in the case of clade *5* and *9*, to group those haplotypes that form a star-like pattern (*5i* and *9i*).

We found many polytomy nodes, as well as several circle structures within individual *Ia* clades in the MJ network (Fig 2), and many nodes with weak bootstrap support within individual *Ia* clades in the ML tree (not shown) (Fig 1). Furthermore, we discovered three polytomies (node 1–3) among the *Ia* clades. In the MJ network, node 1 and node 3 were clear polytomy nodes, whereas node 2 matched a polytomy rather than bifurcation. Node 1 corresponds to the common origin of all *Ia* isolates and splits into the lineage of clade *1*, the lineage of clades *2*–*5*, and the lineage of clades *6*–*9*. Node 2 originated from node 1 and it splits into the lineage of clade *2*, the lineage of clades *3* and *4*, and the lineage of clade *5*; by contrast, node 3 originated from the bifurcating node Y and splits into the lineage of clade *7*, the lineage of clade *8*, and the lineage of clade *9*. Furthermore, the low ML bootstrap support value of the three nodes BC1–3 and the fact that these nodes were only present in the ML tree and not in the MJ nor NN networks (Figs 1a, 1b and 2), supported the assumption of a polytomy.

The nucleotide diversity (π) of the total European *Ia* population (number of used sequences: 651) was 0.029 π. The nucleotide diversity of an individual *Ia* clade ranged from 0.005 (clade *7*) to 0.009 (clade *9*). By comparison, the nucleotide diversity of the Danish *Ia* population (number of used sequences: 236) was 0.014 π, and the nucleotide diversity of the German *Ia* population (number of used sequences: 296) was 0.024 π.

The number of haplotypes that were included within an individual *Ia* clade varied from two (clade *3*) to 158 (clade *9*). The mean period of occurrence of a single *Ia* haplotype was 1.482 years (standard deviation (σ) = 0.55 years) (S1 Table). A striking exception was one haplotype of clade *6* (number of haplotypes: 140) that included a German isolate from 1990 (V01-90he) and a French isolate from 1971 (FR-0771), which was thus re-isolated 19 years after its first isolation. This exceptional case has led us to exclude this haplotype from the calculation of the mean period of occurrence of the *Ia* haplotypes. In addition, as this haplotype is a member of clade 6, we have excluded this clade from the calculation of the mean period of occurrence of the *Ia* clades. The mean period of occurrence of a *Ia* clade was 11.4 years (σ = 6.5 years) (Fig 2 and S1 Table). Notably, German *Ia* haplotypes from 2005 to 2015 fall into four distinct *Ia* clades (*4*, *5*, *8*, and *9*), whereas Danish *Ia* haplotypes from 1999 to 2009 fall into two *Ia* clades (*5* and *9*). By comparison, the mean value was 4.7 *Ia* clades per calendar year (σ = 1.658 per calendar year) for the entire dataset, calculated over the last 20 years (1996–2015). On the basis of the phylogeographic pattern of the *Ia* population (Fig 2), clades *1*, *3*, *4*, and *6* indicate a trend toward Germany and France, whereas clades 2 and 7 suggest a trend toward Germany and Denmark. In case of clades *8* and *9*, the phylogeographic pattern was particularly difficult to interpret, as each of them included isolates from an especially large number of European countries (Fig 2 and S1 Table). While the isolates of clade *8* were from Austria, the Czech Republic, France, Germany, Italy, Poland, and Switzerland, the isolates of clade *9* were from Austria, Denmark, France, Germany, Italy, Poland, Slovenia, and Switzerland. However, both clades were dominated by German isolates. By contrast, although clade *5* also includes several German isolates and an isolate from the United Kingdom, this clade exhibited a clear trend toward Denmark (Fig 2).

Furthermore, 4.02% of the *Ia* haplotypes appeared in more than one European country (nine haplotypes of clade *9*, three of clade *8*, one of clade *6*, and three of clade *5*) (Fig 2 and S1 Table). The most widespread *Ia* haplotype—haplotype number 161, occurring in Germany, France, Poland, and Switzerland—was located very close to the center of clade *9*, which was the most widespread *Ia* clade with haplotypes isolated from seven European countries: Austria, France, Germany, Poland, Italy, Slovenia, and Switzerland (Fig 2 and S1 Table). In addition, this clade contained the highest number of *Ia* haplotypes that occurred in more than one country (Fig 1 and S1 Table).

**Fig 3:**
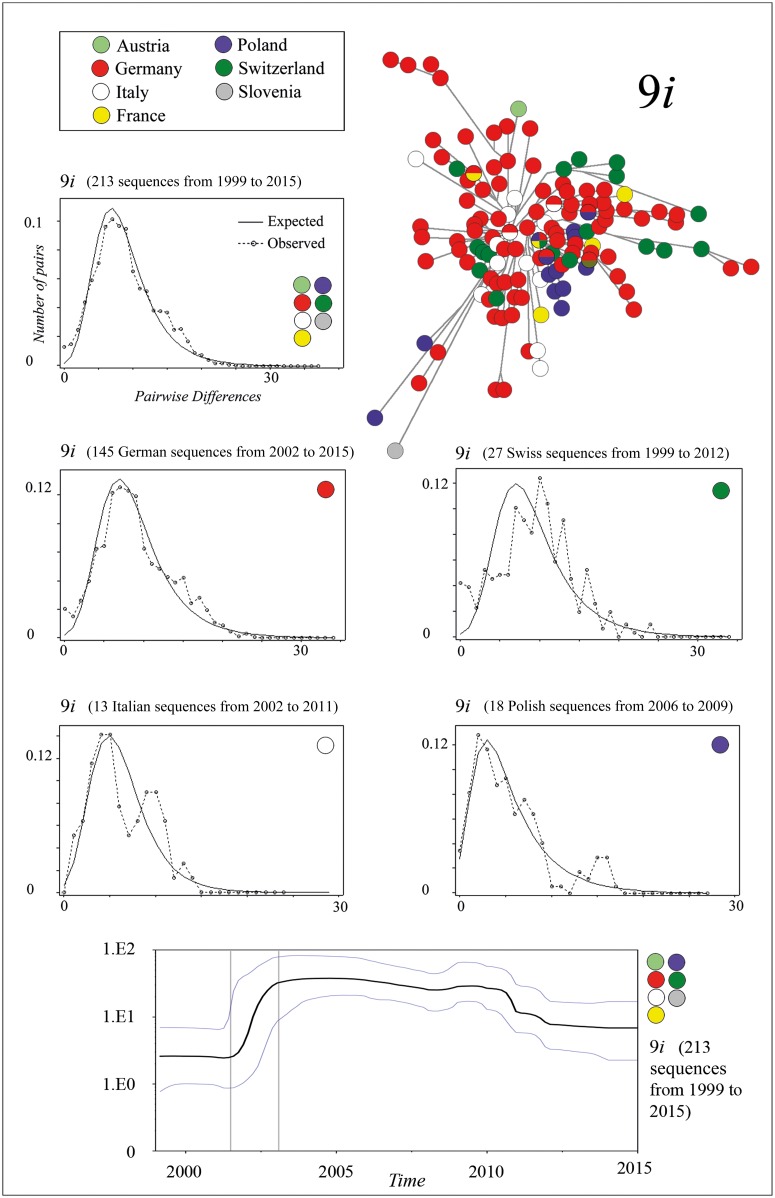
Mismatch distributions and Bayesian skyline plot of 9*i* sequences. The mismatch distribution is based on the complete *G* gene sequence and is calculated separately for the following: 213 *9i* sequences from Austria, France, Germany, Italy, Poland, Switzerland, and Slovenia, collected between 1999 and 2015; 145 German *9i* sequences from 2002 to 2015; 27 Swiss *9i* sequences from 1999 to 2012; 13 Italian *9i* sequences from 2002 to 2011; and 18 Polish *9i* sequences from 2006 to 2009. Table 1 lists the respective value of the raggedness index (r), nucleotide diversity (PI), Tajima’s D, and Fu’s Fs of each dataset. The Bayesian skyline plot shows changes of the 9*i* population size between 1999 and 2015. The plot was generated using all 213 9*i* sequences (complete *G* gene sequence). X axis: time in years, Y axis: population size. The middle solid line is the median estimate, and the area between the blue lines shows the 95% highest probability density (HPD).

**Fig 4:**
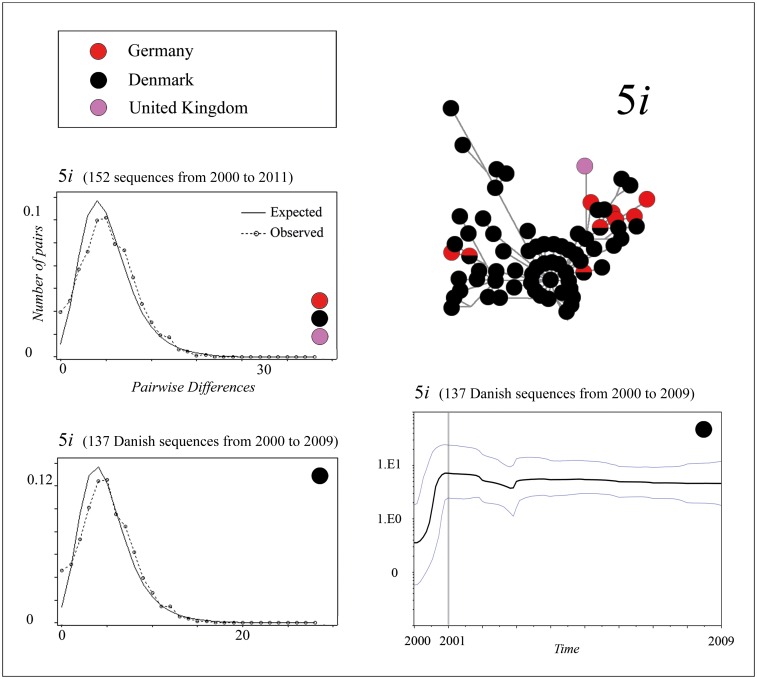
Mismatch distributions and Bayesian skyline plot of 5*i* sequences. The mismatch distribution is based on the complete *G* gene sequence and is calculated separately for the following: 152 *5i* sequences from Denmark, Germany, and the United Kingdom, collected between 2000 and 2011; and 137 Danish *5i* sequences from 2000 to 2009. Table 1 lists the respective values of the raggedness index (r), nucleotide diversity (PI), Tajima’s D, and Fu’s Fs of each dataset. The Bayesian skyline plot shows changes of the 5*i* population size between 2000 and 2009. The plot was generated using 137 Danish 5*i* sequences (complete *G* gene sequence). X axis: time in years, Y axis: population size. The middle solid line is the median estimate, and the area between the blue lines shows the 95% highest probability density (HPD).

We found indications for two instances of large-scale rapid population growth during the evolution of the *Ia* lineage. One of these events took place in the 9*i* population of clade *9*, and the other event occurred in the *5i* population of clade *5*. This finding was based on two major star-like patterns in the MJ network (Fig 2). During these events, the substitution rate was 7.813 × 10^−4^ nucleotides per site per year for the 5*i* haplotypes, and 5.651 × 10^-4^nucleotides per site per year for the 9*i* haplotypes. These potential demographic expansions were also indicated through a Bayesian skyline plot and a mismatch distribution via a unimodal curve (Figs 3 and 4). On the basis of the Bayesian skyline plot, this rapid increase in the population size of thlle 5*i* population occurred between approximately 2000 and 2001, and in the case of the 9*i* population, it occurred between approximately 2001 and 2003. Moreover, we confirmed a low value for the raggedness index and statistically significant negative values in tests for Tajima’s D and Fu’s Fs for both datasets (Table 1). This was also the case when we examined separately either the Danish 5*i* haplotypes or the German 9*i* haplotypes (Figs 3 and 4, Table 1). However, when we calculated the mismatch distribution of clade *9i* haplotypes exclusively from Switzerland, Italy, or Poland (Fig 3), and also of the haplotypes from *5ii*, *8ii*, *8iii*, and *9ii* (graphs not shown), the data only poorly fit the unimodal curve. Instead of a smooth unimodal shape, these curves exhibit a more ragged shape. Accordingly, the raggedness index was significantly increased and a negative non-significant *P* value was also obtained in tests for Tajima’s D and Fu’s Fs (Table 1). By contrast, the multimodal curve of haplotypes from clade *2* had no similarity to a unimodal curve (graph not shown). In case of clades *1*, *3*, *4*, *6*, and *7*, and *8iii* and *9iii*, we did not perform a demographic expansion analysis because of the small number of different haplotypes in those clades.

**Table 1:** Parameters of the analysis of a rapid population growth based on the complete *G* gene sequence data.

Clade and subset membership, as well as sampling period	Number of used sequences (n)	Nucleotide diversity (PI)	Tajima’s D	Fu’s Fs	Raggedness index (r)
5*i* (2000–2011)	152	0.00349	-2.36505**	-73.024	0.0051
5*i* Denmark (2000–2009)	137	0.00333	-2.16059*	-63.686	0.0057
9*i* (1999–2015)	213	0.00583	-2.48568***	-149.539	0.0030
9*i* Germany (2002–2015)	145	0.00584	-2.10903**	-72.958	0.0042
9*i* Italy (2002–2011)	13	0.00407	-1.25092°	-8.124	0.0155
9*i* Poland (2006–2009)	18	0.00354	-1.76634°°	-4.204	0.0120
9*i* Switzerland (1999–2012)	27	0.00609	-0.96486°	-2.551	0.0116

*P<0.05

**P<0.01

***P<0.001

°*P*>0.10 (not significant)

°°0.10>*P*>0.05 (not significant)

## Discussion

Since the first *Ia* isolate was detected in France in 1971, the *Ia* population has become widely distributed in Europe, especially where rainbow trout is intensively produced in freshwater aquaculture [9]. This extensive spread is clearly confirmed by our phylogenetic results. First, the *Ia* lineage underwent a considerable phylogenetic radiation, since we were able to divide this subgenogroup into nine *Ia* clades (Fig 2). Normally, such radiation is based on different local scenarios of selection and genetic drift resulting from the spread events. In addition, isolate X may even indicate—as a tenth *Ia* clade—the existence of further clades which remain, however, undetected using this dataset. Secondly, each of the three polytomies (nodes 1–3) (Fig 2) that were found among the *Ia* clades could be an indicator for a rapid radiation, as they reflect a simultaneous spread from one single place to several places. For example, in the case of even a single delivery of VHS-infected trout, one single *Ia* haplotype can be spread to various regions of Europe. However, this is only true, if these polytomies are hard polytomies and not the result of a potential taxon sampling bias in our dataset. Third, the Europe-wide distribution of clades *8* and *9*, in particular, indicates a strong mixing of *Ia* isolates through the trout trade. Clade *8* includes isolates from seven different European countries, and clade *9* from eight (Fig 2 and S1 Table). Finally, the indications for the two large-scale rapid population growth events in the *Ia* population could also point to, at least temporally, a sudden extensive demographic expansion, which is normally the result of the spatial spread of a species [42, 43].

However, a clear reconstruction of the spread routes of ancestral lineages of individual *Ia* clades was largely impossible. The reason for that was that most *Ia* clades include several nodes with weak ML bootstrap support (not shown), a result of the low resolving power of mutational differences among their closely related haplotypes, and apparently due to homoplasy (parallel or back mutation events) [33]. This poor phylogenetic resolution was also apparent in the MJ network through the many polytomy structures, and several circle structures within the *Ia* clades (Fig 2). Furthermore, this reconstruction was additionally complicated by an almost complete absence of a country-specific distribution for most of the *Ia* clades (Fig 2). As mentioned above, it seems that the *Ia* isolates were permanently and complexly changed due to the Europe-wide mixing of *Ia* isolates via the trout trade. And finally, the reconstruction of the spread routes was further complicated, by a sampling bias in our dataset. Although an extensive dataset of *Ia* isolates was used here, spatiotemporal sampling bias may exist in the case of *Ia* isolates obtained from Austria, the Czech Republic, France, Georgia, Italy, Poland, Slovenia, Switzerland, and the United Kingdom, and from Germany and Denmark before 1996 (S1 Fig). This is possible, as outbreak samples from European trout farms (let alone from farms with latent infections) were collected using different levels of strictness depending on individual national surveillance of VHS. Therefore, we believe that this study may represent an incomplete pan-European picture of the country-specific distribution for most *Ia* clades. This circumstance has also contributed to our decision to largely avoid the reconstruction of the spread routes for the ancestral lineages of these *Ia* clades. For example, we consciously decided against speculating on the place of origin of the *Ia* lineage. This place might be obscured by an extensive Europe-wide spread of the *Ia* lineage shortly after its origin. On the one hand, this could explain the polytomy structure of node 1, provided that it reflects a hard polytomy(Figs 1c and 2). But in view of the above mentioned potential sampling bias, this polytomy cannot be seen as an argument for a real hard polytomy. On the other hand, the isolates that cluster closest to this node 1 (clades *1*, *2*, and *6*) were from different European countries (Denmark, Germany, and France) (Fig 2). In addition, the determination of the place of origin is further complicated because our dataset exhibits a spatiotemporal sampling bias of *Ia* isolates from the period when the first *Ia* isolates were collected (S1 Fig).

Due to the particularly high spatiotemporal density of Danish and German *Ia* isolates in our dataset from approximately the last 20 years, we particularly focused our phylogenetic analysis on those two countries during this period. Thus, in comparison to the German situation, we observed an instance of rare gene flow of *Ia* isolates between Denmark and other European countries. This was supported by the following three findings. (1) Haplotypes of the dominant Danish clade (clade *5*)—the clade with the largest number of different Danish haplotypes—were almost exclusively detected in Denmark, whereas many haplotypes of clade *9*—the clade with the largest number of different German haplotypes—were also found outside Germany in several other European countries (Fig 1 and S2 Table). In addition, we found no isolate of clade *9* in Denmark, even though this was the most widespread clade in Europe (occurring in nine different European countries) (Fig 1 and S1 Table). In contrast, the clear dominance of Danish isolates in clade *5* (Fig 2 and S1 Table) strongly indicates a Danish origin of this clade, and therefore it can be assumed that the ancestral lineage of the German and British isolates of clade *5* was introduced from Denmark. This conclusion also agrees with the findings of Kahns and his colleagues [22]. (2) Danish isolates fall into only four of the nine *Ia* clades detected, whereas German isolates fall into all nine (Fig 2 and S1 Table). Accordingly, the nucleotide diversity of the German *Ia* population (304 used sequences) was almost more than twice as high as the nucleotide diversity of the Danish *Ia* population (244 used sequences) (not shown). (3) Danish haplotypes that were also detected outside Denmark were only found in Germany, whereas German haplotypes that were detected outside Germany were also found in several other European countries (Austria, Denmark, France, Italy, the Netherlands, Poland, and Switzerland) (Fig 2 and S1 Table).

This relatively rare cross-border exchange of *Ia* isolates between Denmark and other European countries very likely reflects the strict control of export and import of rainbow trout in Denmark. As Danish fish farms produce fish mainly under tight economic pressure and their production volumes are mostly large, the potential economic impact of a VHS outbreak is substantial. Therefore, since the 1960s, several extensive sanitation and eradication programs have been implemented to control VHS, and since the spring of 2009, no VHS has been detected in any Danish fish farm [22]. In contrast to Danish trout farming conditions, approximately 95% of all German trout farms are very small (annual production volume of <5 metric tons; Survey and Diagnosis 2014, http://www.eurl-fish.eu/Activities/survey_and_diagnosis), and their trout production is intended more for the local market or even for self-sufficiency. The smaller economic weight of rainbow trout production in Germany could be one reason for this less strict control.

Given that the rapid population growth of the 5*i* population took place exclusively in Denmark between approximately 2000 and 2001 (Fig 4) (provided that our analysis reflects a demographic expansion and not a selective sweep), it therefore occurred during the final period of the Danish VHS-eradication program [22]. This was surprising as such an event can point, under certain circumstances, to a biosecurity gap; for example, when dealing with practices of aquaculture or fish trade for farming and restocking purposes. The underlying idea is that careless handling of VHSV-infected fish may suddenly result in an increased spread rate of the virus, which is normally the basis for a sudden demographic expansion event [45]. However, such an increased spread rate can also be the result of either a sudden non-human-mediated change in the natural environment (e.g., changes in the climate), or a successful evolutionary adaptation towards increased fitness that may have acted within these circulating VHSV infections. Seen in this light, we believe that the causative factor of this Danish demographic expansion event may be of particular interest in follow-up studies.

In the case of the potential rapid population growth of the 9*i* population, we think that Germany was a crucial place for its emergence, at least from approximately 2001–2003 (Fig 3). Nevertheless, we found 9*i* isolates in eight further European countries (Fig 1). Accordingly, their participation in the population growth was not excludable, because of the mentioned sampling bias of *Ia* isolates in our dataset. In particular, the role of Italy, Poland, and Switzerland was at issue, as their jagged mismatch curves still exhibited a discernible unimodal shape that may indicate their participation (Fig 3). In the case of Switzerland, however, we know that the trout farming industry did not sell life trout abroad during this period; instead, they bought many life trout from surrounding countries. We only exclude Danish participation, as our dataset comprised no Danish 9*i* isolates, even though our dataset has a high spatiotemporal density of Danish isolates from that period.

At first sight, this Europe-wide spread and mixing of *Ia* isolates seems to be surprisingly inconsistent with another outcome of this study, namely that only about 4% of the *Ia* haplotypes occurred in more than one European country (S1 Table). However, we think that this can most probably be explained by the observed high substitution rate of VHSV [13, 17]. Our dataset indicates that the mean period of occurrence of a single *Ia* haplotype is typically not longer than one calendar year. Furthermore, distinct isolates from the same VHS outbreak occasionally fall into different but very closely related haplotypes (e.g., isolates V43-14bb, V44-14bb, and V45-14bb) (S1 Table). However, the observation that one haplotype in our dataset (number 140) was re-isolated (as isolate V01-90he) 19 years after its first isolation (as isolate FR-0771) can be best explained by laboratory contamination or a confusion event.

Our dataset suggests that *Ia* clades become extinct after a period, since the mean period of occurrence of a clade is approximately 11 years (Fig 2). We consider that this cyclic pattern of newly arising formations and the disappearance of clades have been mainly caused by anthropogenic influences such as trout farmers and traders, since the host of the *Ia* population has mainly been the European farmed rainbow trout. Approximately 95% of the *Ia* isolates of our dataset are from farmed rainbow trout. This close ecological interaction is supported by the fact that *Ia* isolates have, in contrast to isolates from other subgenogroups of the genogroup *I* (*Ib*–*Ie*) and the genogroups *II*–*IV*, a particularly high virulence for farmed rainbow trout [12, 46]. We believe that careless breeding and trade practices when dealing with VHS-infected fish have unintentionally supported the geographical spread of the virus and the establishment of new clades, while sanitation and eradication measures have led to their extinction. We also largely exclude a natural host’s adaptation toward resistance against the virus. This is because farmed animal stocks frequently suffer from weaker resistance against infectious disease and often have a lower potential for evolutionary resistance adaptation in comparison to their wild ancestors. Furthermore, housing and trading conditions make farmed stocks particularly vulnerable to the spread of pathogens. The main reasons for this are: (1) the loss of variation at resistance loci because of founder effects that result from domestication and intentional breeding events [47–49]; (2) a decreased chance of successfully adapting towards disease resistance due to artificial mating systems and a consequent relaxation of natural selection [50, 51]; (3) the danger of pleiotropic effects (negative side effects) from intentional breeding that can negatively influence disease resistance [52]; (4) factory farming practices that can lead to a stress-induced weak immune system due to artificial housing conditions and high population density [50, 53]; and last but not least, (5) a high population density that can additionally increase virus transmissibility [54].

## Supporting Information

S1 Fig Chronology of the geographic collection of 422 *Ia* isolates. (DOCX)

S1 Table Data on the 717 VHSV isolates used in this study. The samples highlighted in white are the extended samples obtained from GenBank. The table includes the NCBI accession number, Fispathogens.eu database (EU-FP) number, name of isolate, date of collection, site of collection, host species (*wild fish; **feral fish; no asterisk, characterized as a farmed fish), and phylogenetic classification (*Ia*: haplotype-subgenogroup-clade; *I(unclassified)*, *Ib*, *Ic*, *Id*, and *Ie*: haplotype-subgenogroup; and *II*, *III*, *IV*: genogroup). Superscripts in parentheses mark corrections with respect to the original protocol in GenBank of the respective accession number: 1) corrections to the name of host species; 2) to the year of isolation; 3) to the name of isolate.
(DOCX)

S2 Table Primer sequences. (DOCX)

## Abbreviations

*G* gene: *glycoprotein* gene
VHS: viral hemorrhagic septicemia
VHSV: VHS virus
