# Supplementary figures and images for: Phylogeny of the Viral Hemorrhagic Septicemia Virus in European Aquaculture
Source: PLoS One. 2016 Oct 19;11(10):e0164475. doi: 10.1371/journal.pone.0164475 (PMC5070809; doi:10.1371/journal.pone.0164475)

**S1 Fig. Chronology of the geographic collection of 422 *Ia* isolates.**


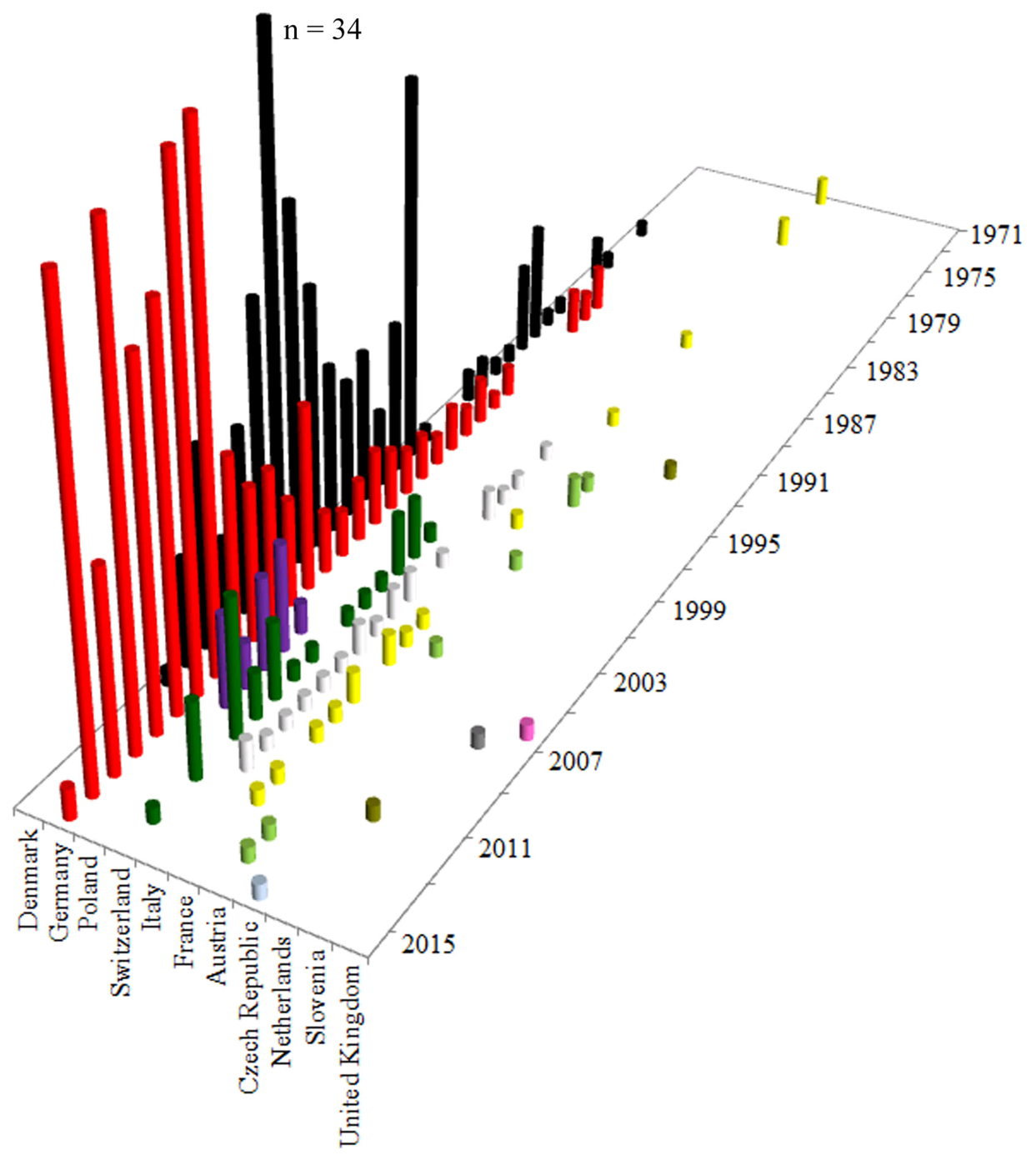

Supplement: S1 Fig — (DOCX) [file pone.0164475.s001.docx]
