# Supplementary material for: Phylogeny of the Viral Hemorrhagic Septicemia Virus in European Aquaculture
Source: PLoS One. 2016 Oct 19;11(10):e0164475. doi: 10.1371/journal.pone.0164475 (PMC5070809; doi:10.1371/journal.pone.0164475)
Supplement: S1 Table — The samples highlighted in white are the extended samples obtained from GenBank. The table includes the NCBI accession number, Fispathogens.eu database (EU-FP) number, name of isolate, date of collection, site of collection, host species (*wild fish; **feral fish; no asterisk, characterized as a farmed fish), and phylogenetic classification (Ia: haplotype-subgenogroup-clade; I(unclassified), Ib, Ic, Id, and Ie: haplotype-subgenogroup; and II, III, IV: genogroup). Superscripts in parentheses mark corrections with respect to the original protocol in GenBank of the respective accession number: 1) corrections to the name of host species; 2) to the year of isolation; 3) to the name of isolate. (DOCX) [file pone.0164475.s002.docx]

**S1 Table.** **Data on the 708 VHSV isolates used in this study.** The samples highlighted in white are the extended samples obtained from GenBank. The table includes the NCBI accession number, Fispathogens.eu database (EU-FP) number, name of isolate, date of collection, site of collection, host species (*wild fish; **feral fish; no asterisk, characterized as a farmed fish), and phylogenetic classification (*Ia*: haplotype-subgenogroup-subclade; *I(unclassified)*, *Ib*, *Ic*, *Id*, and *Ie*: haplotype-subgenogroup; and *II*, *III*, *IV*: genogroup). Superscripts in parentheses mark corrections with respect to the original protocol in GenBank of the respective accession number: 1) corrections to the name of host species; 2) to the year of isolation; 3) to the name of isolate.

| ***NCBI accession number*** | ***EU-FP number*** | ***Name of isolate*** | ***Date of collection*** | ***Site of collection*** | ***Host species*** | ***Phylogenetic classification*** |
| --- | --- | --- | --- | --- | --- | --- |
| AF345857 | FP.VHSV.35 | DK-F1 | 1962 | Denmark | *Oncorhynchus mykiss* | 1-I(*unclass*.) |
| Z93412 | FP.VHSV.10 | Hededam | 1970 | Denmark | *Oncorhynchus mykiss* | 3-I(*unclass*.) |
| U28798 | FP.VHSV.10 | Hededam | 1970 | Denmark | *Oncorhynchus mykiss ^1)^* | 2-I(*unclass*.) |
| LN876781 | FP.VHSV.789 | DK-2075 | 1977 | Denmark | *Oncorhynchus mykiss* | 4-Ic |
| LN876782 | FP.VHSV.790 | DK-2079 | 1977 | Denmark | *Oncorhynchus mykiss* | 125-Ia-10 |
| LN876783 | FP.VHSV.791 | DK-2149 | 1978 | Denmark | *Oncorhynchus mykiss* | 5-Ic |
| LN876784 | FP.VHSV.792 | DK-2189 | 1978 | Denmark | *Oncorhynchus mykiss* | 4-Ic |
| LN876785 | FP.VHSV.793 | DK-2283 | 1979 | Denmark | *Oncorhynchus mykiss* | 4-I(*unclass*.) |
| LN876786 | FP.VHSV.794 | DK-2430 | 1980 | Denmark | *Oncorhynchus mykiss* | 62-Ia-10 |
| LN876787 | FP.VHSV.795 | DK-2503 | 1980 | Denmark | *Oncorhynchus mykiss* | 6-Ic |
| LN876788 | FP.VHSV.796 | DK-2568 | 1981 | Denmark | *Oncorhynchus mykiss* | 63-Ia-2 |
| LN876789 | FP.VHSV.797 | DK-2589 | 1981 | Denmark | *Oncorhynchus mykiss* | 63-Ia-2 |
| LN876790 | FP.VHSV.798 | DK-2634 | 1981 | Denmark | *Oncorhynchus mykiss* | 72-Ia-2 |
| AY546585 | FP.VHSV.11 | DK-2835 | 1982 | Denmark | *Oncorhynchus mykiss* | 9-Ic |
| LN876791 | FP.VHSV.799 | DK-3164 | 1984 | Denmark | *Oncorhynchus mykiss* | 73-Ia-2 |
| LN876792 | FP.VHSV.800 | DK-3539 | 1985 | Denmark | *Oncorhynchus mykiss* | 64-Ia-2 |
| X66134 | FP.VHSV.13 | DK-3592B | 1986 | Denmark | *Oncorhynchus mykiss* | 123-Ia-2 |
| LN876793 | FP.VHSV.801 | DK-3555 | 1986 | Denmark | *Oncorhynchus mykiss*** | 64-Ia-2 |
| LN876794 | FP.VHSV.802 | DK-3612 | 1986 | Denmark | *Oncorhynchus mykiss* | 7-Ic |
| LN876795 | FP.VHSV.803 | DK-3647 | 1986 | Denmark | *Oncorhynchus mykiss* | 15-Ia-2 |
| LN876796 | FP.VHSV.804 | DK-3648 | 1986 | Denmark | *Oncorhynchus mykiss* | 15-Ia-2 |
| LN876797 | FP.VHSV.805 | DK-3654 | 1986 | Denmark | *Oncorhynchus mykiss* | 13-Ia-5 |
| LN876798 | FP.VHSV.806 | DK-3768 | 1986 | Denmark | *Oncorhynchus mykiss* | 64-Ia-2 |
| LN876799 | FP.VHSV.807 | DK-3769 | 1986 | Denmark | *Oncorhynchus mykiss* | 64-Ia-2 |
| LN876800 | FP.VHSV.808 | DK3856 | 1986 | Denmark | *Oncorhynchus mykiss* | 15-Ia-2 |
| AY546587 | FP.VHSV.17 | DK-3971 | 1987 | Denmark | *Oncorhynchus mykiss* | 15-Ia-2 |
| AY546586 | FP.VHSV.14 | DK-3946 | 1987 | Denmark | *Oncorhynchus mykiss* | 14-Ia-2 |
| LN876801 | FP.VHSV.809 | DK-3889 | 1987 | Denmark | *Oncorhynchus mykiss* | 65-Ia-5 |
| LN876802 | FP.VHSV.810 | DK-3902 | 1987 | Denmark | *Oncorhynchus mykiss* | 64-Ia-2 |
| LN876803 | FP.VHSV.811 | DK-3913 | 1987 | Denmark | *Oncorhynchus mykiss* | 74-Ia-5 |
| LN876804 | FP.VHSV.817 | DK-3925 | 1987 | Denmark | *Oncorrhynchus mykiss* | 83-Ia-2 |
| AF345858 | FP.VHSV.19 | DK-5131 | 1988 | Denmark | *Oncorhynchus mykiss* | 8-Ic |
| AF345859 | FP.VHSV.21 | DK-5151 | 1988 | Denmark | *Oncorhynchus mykiss* | 13-Ia-5 |
| AY546589 | FP.VHSV.23 | DK-5727 | 1989 | Denmark | *Oncorhynchus mykiss* | 16-Ia-2 |
| AY546590 | FP.VHSV.24 | DK-5740 | 1990 | Denmark | *Oncorhynchus mykiss* | 16-Ia-2 |
| AY546591 | FP.VHSV.25 | DK-5741 | 1990 | Denmark | *Oncorhynchus mykiss* | 16-Ia-2 |
| AY546592 | FP.VHSV.224 | DK-6045 | 1991 | Denmark | *Oncorhynchus mykiss* | 17-Ia-5 |
| AY546593 | FP.VHSV.225 | DK-6137 | 1991 | Denmark | *Oncorhynchus mykiss* | 18-Ia-5 |
| AY546594 | FP.VHSV.31 | DK-7380 | 1994 | Denmark | *Oncorhynchus mykiss* | 19-Ia-5;  20-Ia-5 |
| AY546595 | FP.VHSV.33 | DK-7974 | 1995 | Denmark | *Oncorhynchus mykiss* | 21-Ia-5 |
| LN876805 | FP.VHSV.679 | DK-7831 | 1995 | Denmark | *Oncorhynchus mykiss* | 75-Ia-5;  76-Ia-5 |
| LN876806 | FP.VHSV.680 | DK-7832 | 1995 | Denmark | *Oncorhynchus mykiss* | 19-Ia-5 |
| LN876807 | FP.VHSV.681 | DK-7839 | 1995 | Denmark | *Oncorhynchus mykiss* | 19-Ia-5 |
| LN876808 | FP.VHSV.816 | DK-7840 | 1995 | Denmark | *Oncorhynchus mykiss* | 87-Ia-5 |
| LN876809 | FP.VHSV.683 | DK-7847-3 | 1995 | Denmark | *Oncorhynchus mykiss* | 87-Ia-5 |
| LN876810 | FP.VHSV.684 | DK-7886 | 1995 | Denmark | *Oncorhynchus mykiss* | 87-Ia-5 |
| LN876811 | FP.VHSV.685 | DK-7887 | 1995 | Denmark | *Oncorhynchus mykiss* | 96-Ia-5 |
| LN876812 | FP.VHSV.686 | DK-7888 | 1995 | Denmark | *Oncorhynchus mykiss* | 98-Ia-5 |
| LN876813 | FP.VHSV.687 | DK7890 | 1995 | Denmark | *Oncorhynchus mykiss* | 87-Ia-5 |
| LN876814 | FP.VHSV.688 | DK-7892 | 1995 | Denmark | *Oncorhynchus mykiss* | 96-Ia-5 |
| LN876815 | FP.VHSV.689 | DK-7953 | 1995 | Denmark | *Oncorhynchus mykiss* | 87-Ia-5 |
| LN876816 | FP.VHSV.690 | DK-7979-7 | 1995 | Denmark | *Oncorhynchus mykiss* | 19-Ia-5 |
| LN876817 | FP.VHSV.691 | DK-7996 | 1995 | Denmark | *Oncorhynchus mykiss* | 96-Ia-5 |
| LN876818 | FP.VHSV.692 | DK-7997 | 1995 | Denmark | *Oncorhynchus mykiss* | 98-Ia-5 |
| LN876819 | FP.VHSV.693 | DK-8006-1 | 1995 | Denmark | *Oncorhynchus mykiss* | 110-Ia-5 |
| LN876820 | FP.VHSV.694 | DK-8032 | 1995 | Denmark | *Oncorhynchus mykiss* | 76-Ia-5 |
| LN876821 | FP.VHSV.695 | DK-8047 | 1995 | Denmark | *Oncorhynchus mykiss* | 110-Ia-5 |
| LN876822 | FP.VHSV.696 | DK-8154 | 1995 | Denmark | *Oncorhynchus mykiss* | 76-Ia-5 |
| LN876823 | FP.VHSV.697 | DK-8282 | 1995 | Denmark | *Oncorhynchus mykiss* | 110-Ia-5 |
| LN876824 | FP.VHSV.698 | DK-8314 | 1995 | Denmark | *Oncorhynchus mykiss* | 117-Ia-5 |
| LN876825 | FP.VHSV.699 | DK-8322 | 1995 | Denmark | *Oncorhynchus mykiss* | 119-Ia-5 |
| LN876826 | FP.VHSV.700 | DK-8325-3 | 1995 | Denmark | *Oncorhynchus mykiss* | 383-Ia-5 |
| LN876827 | FP.VHSV.701 | DK-8326-3 | 1995 | Denmark | *Oncorhynchus mykiss* | 384-Ia-5 |
| LN876828 | FP.VHSV.703 | DK-8329 | 1995 | Denmark | *Oncorhynchus mykiss* | 122-Ia-5 |
| AY546597 | FP.VHSV.263 | DK-9695377 | 1996 | Denmark | *Oncorhynchus mykiss* | 22-Ia-2 |
| LN876829 | FP.VHSV.704 | DK-9695056 | 1996 | Denmark | *Oncorhynchus mykiss* | 77-Ia-5 |
| LN876830 | FP.VHSV.705 | DK-9695232 | 1996 | Denmark | *Oncorhynchus mykiss* | 81-Ia-5 |
| LN876831 | FP.VHSV.706 | DK-9695279 | 1996 | Denmark | *Oncorhynchus mykiss* | 81-Ia-5 |
| LN876832 | FP.VHSV.707 | DK-9695343 | 1996 | Denmark | *Oncorhynchus mykiss* | 81-Ia-5 |
| LN876833 | FP.VHSV.713 | DK-9695505 | 1996 | Denmark | *Oncorhynchus mykiss* | 91-Ia-2 |
| LN876834 | FP.VHSV.708 | DK-9695375 | 1996 | Denmark | *Oncorhynchus mykiss* | 93-Ia-2 |
| LN876835 | FP.VHSV.710 | DK-9695379 | 1996 | Denmark | *Oncorhynchus mykiss* | 213-Ia-7 |
| LN876836 | FP.VHSV.711 | DK-9695489 | 1996 | Denmark | *Oncorhynchus mykiss* | 214-Ia-7; 215-Ia-7 |
| LN876837 | FP.VHSV.712 | DK-9695491 | 1996 | Denmark | *Oncorhynchus mykiss* | 105-Ia-5 |
| AY546598 | FP.VHSV.264 | DK-9795568 | 1997 | Denmark | *Oncorhynchus mykiss* | 23-Ia-5 |
| LN876838 | FP.VHSV.718 | DK-9795242-1 | 1997 | Denmark | *Oncorhynchus mykiss* | 211-Ia-7 |
| LN876839 | FP.VHSV.714 | DK-9795045 | 1997 | Denmark | *Oncorhynchus mykiss* | 78-Ia-5 |
| LN876840 | FP.VHSV.715 | DK-9795141 | 1997 | Denmark | *Oncorhynchus mykiss* | 78-Ia-5 |
| LN876841 | FP.VHSV.717 | DK-9795180 | 1997 | Denmark | *Oncorhynchus mykiss* | 88-Ia-2 |
| AY546599 | FP.VHSV.265 | DK-9895024 | 1998 | Denmark | *Oncorhynchus mykiss* | 24-Ia-2 |
| AY546600 | FP.VHSV.266 | DK-9895093 | 1998 | Denmark | *Oncorhynchus mykiss* | 25-Ia-5 |
| LN876842 | FP.VHSV.721 | DK-9795657 | 1998 | Denmark | *Oncorhynchus mykiss* | 66-Ia-5 |
| LN876843 | FP.VHSV.720 | DK-9795569 | 1998 | Denmark | *Oncorhynchus mykiss* | 69-Ia-2 |
| LN876844 | FP.VHSV.722 | DK-9895204 | 1998 | Denmark | *Oncorhynchus mykiss* | 79-Ia-5 |
| LN876845 | FP.VHSV.723 | DK-9895319 | 1998 | Denmark | *Oncorhynchus mykiss* | 28-Ia-5 |
| LN876846 | FP.VHSV.724 | DK-9895390 | 1998 | Denmark | *Oncorhynchus mykiss* | 27-Ia-2 |
| LN876847 | FP.VHSV.725 | DK-9895391 | 1998 | Denmark | *Oncorhynchus mykiss* | 84-Ia-2 |
| LN876848 | FP.VHSV.726 | DK-9895392 | 1998 | Denmark | *Oncorhynchus mykiss* | 212-Ia-7 |
| AY546601 | FP.VHSV.267 | DK-9995007 | 1999 | Denmark | *Oncorhynchus mykiss* | 26-Ia-5 |
| AY546602 | FP.VHSV.268 | DK-9995144 | 1999 | Denmark | *Oncorhynchus mykiss* | 206-Ia-7 |
| AY546603 | FP.VHSV.269 | DK-9895174 | 1999 | Denmark | *Oncorhynchus mykiss* | 27-Ia-2 |
| AY546604 | FP.VHSV.227 | DK-9995361 | 1999 | Denmark | *Oncorhynchus mykiss* | 28-Ia-5 |
| AY546596 | FP.VHSV.262 | DK-9895168 ^3)^ | 1998 ^2)^ | Denmark | *Oncorhynchus mykiss* | 80-Ia-5 |
| LN876849 | FP.VHSV.727 | DK-9995021-1 | 1999 | Denmark | *Oncorhynchus mykiss* | 26-Ia-5 |
| LN876850 | FP.VHSV.730 | DK-9995093 | 1999 | Denmark | *Oncorhynchus mykiss* | 26-Ia-5 |
| LN876851 | FP.VHSV.731 | DK-9995120 | 1999 | Denmark | *Oncorhynchus mykiss* | 26-Ia-5 |
| LN876852 | FP.VHSV.732 | DK-9995140 | 1999 | Denmark | *Oncorhynchus mykiss* | 99-Ia-5 |
| AY546605 | FP.VHSV.7 | DK-200098 | 2000 | Denmark | *Oncorhynchus mykiss* | 207-Ia-2 |
| AY546606 | FP.VHSV.8 | DK-200148 | 2000 | Denmark | *Oncorhynchus mykiss* | 29-Ia-5;  30-Ia-5 |
| AY546607 | FP.VHSV.9 | DK-200149 | 2000 | Denmark | *Oncorhynchus mykiss* | 29-Ia-5;  31-Ia-5 |
| AY546608 | FP.VHSV.1 | DK-200020-1 | 2000 | Denmark | *Oncorhynchus mykiss* | 29-Ia-5 |
| AY546609 | FP.VHSV.2 | DK-200027-3 | 2000 | Denmark | *Oncorhynchus mykiss* | 29-Ia-5 |
| AY546610 | FP.VHSV.3 | DK-200029-1 | 2000 | Denmark | *Oncorhynchus mykiss* | 29-Ia-5 |
| AY546611 | FP.VHSV.4 | DK-200051-1 | 2000 | Denmark | *Oncorhynchus mykiss* | 28-Ia-5 |
| AY546612 | FP.VHSV.5 | DK-200070-4 | 2000 | Denmark | *Oncorhynchus mykiss* | 29-Ia-5 |
| AY546613 | FP.VHSV.6 | DK-200079-1 | 2000 | Denmark | *Oncorhynchus mykiss* | 28-Ia-5 |
| LN876853 | FP.VHSV.733 | DK-200070-9 | 2000 | Denmark | *Oncorhynchus mykiss* | 29-Ia-5 |
| LN876854 | FP.VHSV.735 | DK-200079-2 | 2000 | Denmark | *Oncorhynchus mykiss* | 29-Ia-5 |
| LN876855 | FP.VHSV.736 | DK-201050 | 2001 | Denmark | *Oncorhynchus mykiss* | 82-Ia-5 |
| LN876856 | FP.VHSV.737 | DK-201103-2 | 2001 | Denmark | *Oncorhynchus mykiss* | 85-Ia-5 |
| LN876857 | FP.VHSV.738 | DK-201127 | 2001 | Denmark | *Oncorhynchus mykiss* | 89-Ia-5 |
| LN876858 | FP.VHSV.739 | DK-201128 | 2001 | Denmark | *Oncorhynchus mykiss* | 89-Ia-5 |
| LN876859 | FP.VHSV.740 | DK-201133 | 2001 | Denmark | *Oncorhynchus mykiss* | 94-Ia-5 |
| LN876860 | FP.VHSV.741 | DK-201135 | 2001 | Denmark | *Oncorhynchus mykiss* | 97-Ia-5 |
| LN876861 | FP.VHSV.742 | DK-201173 | 2001 | Denmark | *Oncorhynchus mykiss* | 100-Ia-5 |
| LN876862 | FP.VHSV.743 | DK-201176 | 2001 | Denmark | *Oncorhynchus mykiss* | 102-Ia-5 |
| LN876863 | FP.VHSV.745 | DK-201236 | 2001 | Denmark | *Oncorhynchus mykiss* | 70-Ia-5;  106-Ia-5 |
| LN876864 | FP.VHSV.746 | DK-201249 | 2001 | Denmark | *Oncorhynchus mykiss* | 107-Ia-5 |
| LN876865 | FP.VHSV.747 | DK-201276-3 | 2001 | Denmark | *Oncorhynchus mykiss* | 108-Ia-5 |
| LN876866 | FP.VHSV.748 | DK-201279 | 2001 | Denmark | *Oncorhynchus mykiss* | 70-Ia-5 |
| LN876867 | FP.VHSV.749 | DK-201331 | 2001 | Denmark | *Oncorhynchus mykiss* | 111-Ia-5 |
| LN876868 | FP.VHSV.750 | DK-201337-2 | 2001 | Denmark | *Oncorhynchus mykiss* | 70-Ia-5 |
| LN876869 | FP.VHSV.751 | DK-201338-1 | 2001 | Denmark | *Oncorhynchus mykiss* | 113-Ia-5 |
| LN876870 | FP.VHSV.752 | DK-201339 | 2001 | Denmark | *Oncorhynchus mykiss* | 114-Ia-5 |
| LN876871 | FP.VHSV.753 | DK-201433-10 | 2001 | Denmark | *Oncorhynchus mykiss* | 67-Ia-5 |
| LN876872 | FP.VHSV.754 | DK-202051 | 2002 | Denmark | *Oncorhynchus mykiss* | 70-Ia-5 |
| LN876873 | FP.VHSV.755 | DK-202075 | 2002 | Denmark | *Oncorhynchus mykiss* | 70-Ia-5 |
| LN876874 | FP.VHSV.756 | DK-202076 | 2002 | Denmark | *Oncorhynchus mykiss* | 70-Ia-5 |
| LN876875 | FP.VHSV.757 | DK-202077 3+4 | 2002 | Denmark | *Oncorhynchus mykiss* | 86-Ia-5 |
| LN876876 | FP.VHSV.759 | DK-202078 | 2002 | Denmark | *Oncorhynchus mykiss* | 90-Ia-5 |
| LN876877 | FP.VHSV.760 | DK-202084 | 2002 | Denmark | *Oncorhynchus mykiss* | 92-Ia-5 |
| LN876878 | FP.VHSV.761 | DK-202096 | 2002 | Denmark | *Oncorhynchus mykiss* | 70-Ia-5 |
| LN876879 | FP.VHSV.762 | DK-202102 | 2002 | Denmark | *Oncorhynchus mykiss* | 101-Ia-5 |
| LN876880 | FP.VHSV.763 | DK-202105 | 2002 | Denmark | *Oncorhynchus mykiss* | 103-Ia-5 |
| LN876881 | FP.VHSV.764 | DK-202111 | 2002 | Denmark | *Oncorhynchus mykiss* | 216-Ia-9; 217-Ia-9 |
| LN876882 | FP.VHSV.765 | DK-202117 | 2002 | Denmark | *Oncorhynchus mykiss* | 70-Ia-5 |
| LN876883 | FP.VHSV.766 | DK-202152 | 2002 | Denmark | *Oncorhynchus mykiss* | 70-Ia-5 |
| LN876884 | FP.VHSV.767 | DK-202165 | 2002 | Denmark | *Oncorhynchus mykiss* | 70-Ia-5 |
| LN876885 | FP.VHSV.768 | DK-202181-3 | 2002 | Denmark | *Oncorhynchus mykiss* | 219-Ia-9 |
| LN876886 | FP.VHSV.769 | DK-202262 | 2002 | Denmark | *Oncorhynchus mykiss* | 112-Ia-5 |
| LN876887 | FP.VHSV.770 | DK-202318 | 2002 | Denmark | *Oncorhynchus mykiss* | 70-Ia-5 |
| LN876888 | FP.VHSV.771 | DK-202334 | 2002 | Denmark | *Oncorhynchus mykiss* | 220-Ia-7 |
| LN876889 | FP.VHSV.772 | DK-202369 | 2002 | Denmark | *Oncorhynchus mykiss* | 10-Ia-5 |
| LN876890 | FP.VHSV.773 | DK-202370 | 2002 | Denmark | *Oncorhynchus mykiss* | 116-Ia-5 |
| LN876891 | FP.VHSV.774 | DK-202454 | 2002 | Denmark | *Oncorhynchus mykiss* | 118-Ia-5 |
| LN876892 | FP.VHSV.775 | DK-202455 | 2002 | Denmark | *Oncorhynchus mykiss* | 70-Ia-5;  120-Ia-5 |
| LN876893 | FP.VHSV.776 | DK-202481 | 2002 | Denmark | *Oncorhynchus mykiss* | 121-Ia-5 |
| JQ361160 | FP.VHSV.512 | DK-203120-1 | 2003 | Denmark | *Oncorhynchus mykiss* | 32-Ia-5 |
| LN876894 | FP.VHSV.505 | DK-203214-6 | 2003 | Denmark | *Oncorhynchus mykiss* | 32-Ia-5 |
| LN876895 | FP.VHSV.506 | DK-203214-14 | 2003 | Denmark | *Oncorhynchus mykiss* | 32-Ia-5 |
| LN876896 | FP.VHSV.507 | DK-203214-23 | 2003 | Denmark | *Oncorhynchus mykiss* | 32-Ia-5 |
| LN876897 | FP.VHSV.508 | DK-203214-24 | 2003 | Denmark | *Oncorhynchus mykiss* | 32-Ia-5 |
| LN876898 | FP.VHSV.509 | DK-203214-26 | 2003 | Denmark | *Oncorhynchus mykiss* | 32-Ia-5 |
| LN876899 | FP.VHSV.510 | DK-203214-27 | 2003 | Denmark | *Oncorhynchus mykiss* | 32-Ia-5 |
| LN876900 | FP.VHSV.511 | DK-203214-29 | 2003 | Denmark | *Oncorhynchus mykiss* | 32-Ia-5 |
| LN876901 | FP.VHSV.513 | DK-203355-1 | 2003 | Denmark | *Oncorhynchus mykiss* | 32-Ia-5 |
| LN876902 | FP.VHSV.777 | DK-203016 | 2003 | Denmark | *Oncorhynchus mykiss* | 32-Ia-5 |
| LN876903 | FP.VHSV.778 | DK-203107 | 2003 | Denmark | *Oncorhynchus mykiss* | 70-Ia-5;  71-Ia-5 |
| LN876904 | FP.VHSV.779 | DK-203113 | 2003 | Denmark | *Oncorhynchus mykiss* | 32-Ia-5 |
| LN876905 | FP.VHSV.780 | DK-203114 | 2003 | Denmark | *Oncorhynchus mykiss* | 32-Ia-5 |
| LN876906 | FP.VHSV.781 | DK-203115 | 2003 | Denmark | *Oncorhynchus mykiss* | 32-Ia-5 |
| LN876907 | FP.VHSV.782 | DK-203121 | 2003 | Denmark | *Oncorhynchus mykiss* | 32-Ia-5 |
| LN876908 | FP.VHSV.783 | DK-203122 | 2003 | Denmark | *Oncorhynchus mykiss* | 47-Ia-5 |
| LN876909 | FP.VHSV.784 | DK-203124 | 2003 | Denmark | *Oncorhynchus mykiss* | 95-Ia-5 |
| LN876910 | FP.VHSV.785 | DK-203130 | 2003 | Denmark | *Oncorhynchus mykiss* | 45-Ia-5 |
| LN876911 | FP.VHSV.786 | DK-203173 | 2003 | Denmark | *Oncorhynchus mykiss* | 104-Ia-5 |
| LN876912 | FP.VHSV.787 | DK-203176 | 2003 | Denmark | *Oncorhynchus mykiss* | 32-Ia-5 |
| LN876913 | FP.VHSV.788 | DK-203249 | 2003 | Denmark | *Oncorhynchus mykiss* | 218-Ia-9 |
| LN876914 | FP.VHSV.667 | DK-203286 | 2003 | Denmark | *Oncorhynchus mykiss* | 218-Ia-9 |
| LN876915 | FP.VHSV.668 | DK-203289 | 2003 | Denmark | *Oncorhynchus mykiss* | 218-Ia-9 |
| LN876916 | FP.VHSV.669 | DK-203295 | 2003 | Denmark | *Oncorhynchus mykiss* | 109-Ia-5 |
| LN876917 | FP.VHSV.670 | DK-203330 | 2003 | Denmark | *Oncorhynchus mykiss* | 32-Ia-5 |
| LN876918 | FP.VHSV.671 | DK203349 | 2003 | Denmark | *Oncorhynchus mykiss* | 45-Ia-5 |
| LN876919 | FP.VHSV.672 | DK-203355 | 2003 | Denmark | *Oncorhynchus mykiss* | 32-Ia-5 |
| LN876920 | FP.VHSV.673 | DK-203361 | 2003 | Denmark | *Oncorhynchus mykiss* | 115-Ia-5 |
| LN876921 | FP.VHSV.664 | DK-203305-1 | 2003 | Denmark | *Oncorhynchus mykiss* | 109-Ia-5 |
| LN876922 | FP.VHSV.674 | DK-203464 | 2003 | Denmark | *Oncorhynchus mykiss* | 68-Ia-5 |
| LN876923 | FP.VHSV.675 | DK-203466-2 | 2003 | Denmark | *Oncorhynchus mykiss* | 70-Ia-5 |
| LN876924 | FP.VHSV.676 | DK-203468 | 2003 | Denmark | *Oncorhynchus mykiss* | 70-Ia-5 |
| LN876925 | FP.VHSV.677 | DK-203490 | 2003 | Denmark | *Oncorhynchus mykiss* | 50-Ia-5 |
| LN876926 | FP.VHSV.678 | DK-203491 | 2003 | Denmark | *Oncorhynchus mykiss* | 32-Ia-5 |
| JF681302 | FP.VHSV.602 | DK-204114 | 2004 | Denmark | *Oncorhynchus mykiss* | 32-Ia-5 |
| JF681303 | FP.VHSV.611 | DK-204054 | 2004 | Denmark | *Oncorhynchus mykiss* | 32-Ia-5 |
| JF681304 | FP.VHSV.604 | DK-204188 | 2004 | Denmark | *Oncorhynchus mykiss* | 32-Ia-5 |
| JF681305 | FP.VHSV.603 | DK-204141 | 2004 | Denmark | *Oncorhynchus mykiss* | 32-Ia-5 |
| JF681306 | FP.VHSV.600 | DK-204409 | 2004 | Denmark | *Oncorhynchus mykiss* | 32-Ia-5 |
| JF681307 | FP.VHSV.601 | DK-204142 | 2004 | Denmark | *Oncorhynchus mykiss* | 32-Ia-5 |
| JF681314 | FP.VHSV.599 | DK-204062 | 2004 | Denmark | *Oncorhynchus mykiss* | 37-Ia-5 |
| JF681319 | FP.VHSV.612 | DK-204205 | 2004 | Denmark | *Oncorhynchus mykiss* | 40-Ia-5 |
| JF681320 | FP.VHSV.606 | DK-204072 | 2004 | Denmark | *Oncorhynchus mykiss* | 40-Ia-5 |
| JF681334 | FP.VHSV.608 | DK-204070 | 2004 | Denmark | *Oncorhynchus mykiss* | 45-Ia-5 |
| JF681338 | FP.VHSV.598 | DK-204408-1 | 2004 | Denmark | *Oncorhynchus mykiss* | 47-Ia-5 |
| JF681340 | FP.VHSV.596 | DK-204129 | 2004 | Denmark | *Oncorhynchus mykiss* | 49-Ia-5 |
| JF681341 | FP.VHSV.607 | DK-204040 | 2004 | Denmark | *Oncorhynchus mykiss* | 50-Ia-5 |
| JF681342 | FP.VHSV.610 | DK-204038 | 2004 | Denmark | *Oncorhynchus mykiss* | 50-Ia-5 |
| JF681343 | FP.VHSV.605 | DK-204055 | 2004 | Denmark | *Oncorhynchus mykiss* | 50-Ia-5 |
| JF681347 | FP.VHSV.613 | DK-204022 | 2004 | Denmark | *Oncorhynchus mykiss* | 54-Ia-5 |
| JF681348 | FP.VHSV.597 | DK-204249 | 2004 | Denmark | *Oncorhynchus mykiss* | 55-Ia-5 |
| JF681349 | FP.VHSV.595 | DK-204172 | 2004 | Denmark | *Oncorhynchus mykiss* | 55-Ia-5 |
| JF681357 | FP.VHSV.609 | DK-204157 | 2004 | Denmark | *Oncorhynchus mykiss* | 208-Ia-9 |
| JF681308 | FP.VHSV.584 | DK-205001 | 2005 | Denmark | *Oncorhynchus mykiss* | 32-Ia-5 |
| JF681327 | FP.VHSV.586 | DK-205124-1 | 2005 | Denmark | *Oncorhynchus mykiss* | 40-Ia-5 |
| JF681328 | FP.VHSV.585 | DK-205070 | 2005 | Denmark | *Oncorhynchus mykiss* | 40-Ia-5 |
| JF681326 | FP.VHSV.594 | DK-205159 | 2005 | Denmark | *Oncorhynchus mykiss* | 42-Ia-5 |
| JF681330 | FP.VHSV.591 | DK-205241 | 2005 | Denmark | *Oncorhynchus mykiss* | 42-Ia-5 |
| JF681331 | FP.VHSV.588 | DK-205215 | 2005 | Denmark | *Oncorhynchus mykiss* | 42-Ia-5 |
| JF681332 | FP.VHSV.592 | DK-205297-1 | 2005 | Denmark | *Oncorhynchus mykiss* | 42-Ia-5 |
| JF681333 | FP.VHSV.590 | DK-205090 | 2005 | Denmark | *Oncorhynchus mykiss* | 44-Ia-5 |
| JF681339 | FP.VHSV.582 | DK-205363 | 2005 | Denmark | *Oncorhynchus mykiss* | 48-Ia-5 |
| JF681345 | FP.VHSV.589 | DK-205061 | 2005 | Denmark | *Oncorhynchus mykiss* | 52-Ia-5 |
| JF681346 | FP.VHSV.587 | DK-205416 | 2005 | Denmark | *Oncorhynchus mykiss* | 53-Ia-5 |
| JF681354 | FP.VHSV.593 | DK-205107 | 2005 | Denmark | *Oncorhynchus mykiss* | 59-Ia-5 |
| JF681313 | FP.VHSV.576 | DK-206252 | 2006 | Denmark | *Oncorhynchus mykiss* | 33-Ia-5 |
| JF681309 | FP.VHSV.580 | DK-206136 | 2006 | Denmark | *Oncorhynchus mykiss* | 36-Ia-5 |
| JF681310 | FP.VHSV.583 | DK-206126 | 2006 | Denmark | *Oncorhynchus mykiss* | 36-Ia-5 |
| JF681311 | FP.VHSV.578 | DK-206195 | 2006 | Denmark | *Oncorhynchus mykiss* | 36-Ia-5 |
| JF681312 | FP.VHSV.581 | DK-206137 | 2006 | Denmark | *Oncorhynchus mykiss* | 36-Ia-5 |
| JF681329 | FP.VHSV.579 | DK-206130 | 2006 | Denmark | *Oncorhynchus mykiss* | 43-Ia-5 |
| JF681335 | FP.VHSV.564 | DK-207052 | 2007 | Denmark | *Oncorhynchus mykiss* | 34-Ia-5 |
| JF681315 | FP.VHSV.567 | DK-207022 | 2007 | Denmark | *Oncorhynchus mykiss* | 38-Ia-5 |
| JF681316 | FP.VHSV.566 | DK-207081 | 2007 | Denmark | *Oncorhynchus mykiss* | 38-Ia-5 |
| JF681317 | FP.VHSV.575 | DK-207171 | 2007 | Denmark | *Oncorhynchus mykiss* | 39-Ia-5 |
| JF681318 | FP.VHSV.570 | DK-207227 | 2007 | Denmark | *Oncorhynchus mykiss* | 39-Ia-5 |
| JF681321 | FP.VHSV.573 | DK-207017 | 2007 | Denmark | *Oncorhynchus mykiss* | 41-Ia-5 |
| JF681322 | FP.VHSV.572 | DK-207057 | 2007 | Denmark | *Oncorhynchus mykiss* | 39-Ia-5 |
| JF681337 | FP.VHSV.574 | DK-207193 | 2007 | Denmark | *Oncorhynchus mykiss* | 46-Ia-5 |
| JF681344 | FP.VHSV.756 | DK-207076-2 | 2007 | Denmark | *Oncorhynchus mykiss* | 51-Ia-5 |
| JF681350 | FP.VHSV.568 | DK-207085 | 2007 | Denmark | *Oncorhynchus mykiss* | 56-Ia-5 |
| JF681351 | FP.VHSV.577 | DK-207131 | 2007 | Denmark | *Oncorhynchus mykiss* | 56-Ia-5 |
| JF681355 | FP.VHSV.569 | DK-207023 | 2007 | Denmark | *Oncorhynchus mykiss* | 60-Ia-5 |
| JF681356 | FP.VHSV.571 | DK-207048 | 2007 | Denmark | *Oncorhynchus mykiss* | 61-Ia-5 |
| JF681336 | FP.VHSV.556 | DK-2008-50-48 ^3)^ | 2008 | Denmark | *Oncorhynchus mykiss*** | 34-Ia-5 |
| JF681323 | FP.VHSV.563 | DK-2008-50-223 ^3)^ | 2008 | Denmark | *Oncorhynchus mykiss* | 35-Ia-5 |
| JF681324 | FP.VHSV.560 | DK-2008-50-251 ^3)^ | 2008 | Denmark | *Oncorhynchus mykiss* | 35-Ia-5 |
| JF681325 | FP.VHSV.558 | DK-2008-50-157-1 ^3)^ | 2008 | Denmark | *Oncorhynchus mykiss* | 35-Ia-5 |
| JF681353 | FP.VHSV.559 | DK-2008-50-195-2 ^3)^ | 2008 | Denmark | *Oncorhynchus mykiss* | 58-Ia-5 |
| JF681358 | FP.VHSV.562 | DK-2008-50-308-1 ^3)^ | 2008 | Denmark | *Oncorhynchus mykiss* | 209-Ia-9 |
| JF681359 | FP.VHSV.561 | DK-2008-50-37-1 ^3)^ | 2008 | Denmark | *Oncorhynchus mykiss* | 210-Ia-9 |
| JF681352 | FP.VHSV.555 | DK-2009-50-5-1 ^3)^ | 2009 | Denmark | *Oncorhynchus mykiss* | 35-Ia-5;  57-Ia-5 |
| EU708759 | FP.VHSV.254 | Dfi13-83 | 1983 | Germany | *Oncorhynchus mykiss* | 3-Ia-2 |
| Y18263 | FP.VHSV.254 | Fi13 | 1983 | Germany | *Oncorhynchus mykiss* | 136-Ia-2 |
| LN876927 | FP.VHSV.844 | V03-83nw | 1983 | Germany | *Oncorhynchus mykiss* | 249-Ia-6 |
| LN876928 | FP.VHSV.845 | V01-84nw | 1984 | Germany | unknown | 232-Ia-2 |
| LN876929 | FP.VHSV.846 | V02-84nw | 1984 | Germany | unknown | 126-Ia-2 |
| LN876930 | FP.VHSV.847 | V02-85nw4 | 1985 | Germany | unknown | 128-Ia-2 |
| LN876931 | FP.VHSV.848 | V02-85nw8 | 1985 | Germany | unknown | 129-Ia-2 |
| LN876932 | FP.VHSV.849 | V02-85nw11 | 1985 | Germany | unknown | 127-Ia-2 |
| LN876933 | FP.VHSV.850 | V01-90he | 1990 | Germany | unknown | 140-Ia-6 |
| LN876934 | FP.VHSV.851 | V02-90he | 1990 | Germany | unknown | 245-Ia-6 |
| EU708793 | FP.VHSV.356 | Dsteinbutt | 1991 | Germany | *Scophthalmus maximus* | 9-Ia-5 |
| EU708755 | FP.VHSV.393 | Db359-92 | 1992 | Germany | *Oncorhynchus mykiss* | 156-Ia-8 |
| EU708756 | FP.VHSV.392 | Db416-92 | 1992 | Germany | *Oncorhynchus mykiss* | 157-Ia-8 |
| LN876935 | FP.VHSV.852 | V01-92he | 1992 | Germany | unknown | 233-Ia-7 |
| EU708780 | FP.VHSV.369 | Dsa19-7-93 | 1993 | Germany | *Oncorhynchus mykiss* | 173-Ia-7 |
| LN876936 | FP.VHSV.853 | V01-93he | 1993 | Germany | *unknown* | 234-Ia-7 |
| EU708784 | FP.VHSV.365 | Dsa141-7-94 | 1994 | Germany | *Oncorhynchus mykiss* | 177-Ia-9 |
| LN876937 | FP.VHSV.854 | V01-94he | 1994 | Germany | unknown | 246-Ia-7; 379-Ia-7 |
| EU708774 | FP.VHSV.375 | Dri12-95 | 1995 | Germany | *Oncorhynchus mykiss* | 3-Ic |
| LN876938 | FP.VHSV.855 | V01-95he | 1995 | Germany | unknown | 235-Ia-7 |
| LN876939 | FP.VHSV.856 | V02-95he | 1995 | Germany | unknown | 246-Ia-7 |
| EU708785 | FP.VHSV.364 | Dsa142-96 | 1996 | Germany | *Oncorhynchus mykiss* | 178-Ia-8 |
| LN876940 | FP.VHSV.857 | V01-96he | 1996 | Germany | unknown | 236-Ia-8 |
| LN876941 | FP.VHSV.858 | V02-96he | 1996 | Germany | unknown | 236-Ia-7; 380-Ia-7 |
| EU708735 | FP.VHSV.413 | Dau9-97 | 1997 | Germany | *Oncorhynchus mykiss* | 1-Ic |
| EU708736 | FP.VHSV.412 | Dau10-97 | 1997 | Germany | *Oncorhynchus mykiss* | 2-Ic |
| EU708738 | FP.VHSV.410 | Dau35-97 | 1997 | Germany | *Oncorhynchus mykiss* | 146-Ia-9 |
| EU708748 | FP.VHSV.400 | Dau686-97 | 1997 | Germany | *Oncorhynchus mykiss* | 152-Ia-8 |
| EU708750 | FP.VHSV.398 | Dau1402-97 | 1997 | Germany | *Oncorhynchus mykiss* | 153-Ia-8 |
| EU708740 | FP.VHSV.408 | Dau52-98 | 1998 | Germany | *Oncorhynchus mykiss* | 146-Ia-9 |
| EU708752 | FP.VHSV.396 | Dau1556-98 | 1998 | Germany | *Oncorhynchus mykiss* | 154-Ia-9 |
| EU708754 | FP.VHSV.394 | Daurxs-98 | 1998 | Germany | *Oncorhynchus mykiss x salmon hybrid* | 155-Ia-9 |
| EU708783 | FP.VHSV.366 | Dsa90-98 | 1998 | Germany | *Oncorhynchus mykiss* | 176-Ia-9 |
| EU708739 | FP.VHSV.409 | Dau42-99 | 1999 | Germany | *Oncorhynchus mykiss* | 1-Ia-5 |
| EU708778 | FP.VHSV.371 | Dsa9-99 | 1999 | Germany | *Oncorhynchus mykiss* | 171-Ia-8 |
| EU708779 | FP.VHSV.370 | Dsa12-99 | 1999 | Germany | *Oncorhynchus mykiss* | 172-Ia-9 |
| EU708782 | FP.VHSV.367 | Dsa82-99 | 1999 | Germany | *Oncorhynchus mykiss* | 175-Ia-8 |
| LN876942 | FP.VHSV.859 | V02-99he | 1999 | Germany | *Salmo trutta* x another *Salmonidae* | 381-Ia-9; 382-Ia-9 |
| EU708745 | FP.VHSV.403 | Dau463-00 | 2000 | Germany | *Oncorhynchus mykiss* | 150-Ia-9 |
| EU708746 | FP.VHSV.402 | Dau519-00 | 2000 | Germany | *Oncorhynchus mykiss* | 150-Ia-9 |
| EU708749 | FP.VHSV.399 | Dau1124-00 | 2000 | Germany | *Oncorhynchus mykiss* | 150-Ia-9 |
| EU708777 | FP.VHSV.372 | Dsa05-00 | 2000 | Germany | *Oncorhynchus mykiss* | 170-Ia-8 |
| EU708751 | FP.VHSV.397 | Dau1503-01 | 2001 | Germany | *Oncorhynchus mykiss* | 327-Ia-1 |
| EU708781 | FP.VHSV.368 | Dsa67-01 | 2001 | Germany | *Oncorhynchus mykiss* | 174-Ia-8 |
| EU708791 | FP.VHSV.358 | Dsan152 | 2001 | Germany | *Oncorhynchus mykiss* | 8-Ia-2 |
| EU708753 | FP.VHSV.395 | Daulab | 2001 | Germany | *Oncorhynchus mykiss* | 328-Ia-1 |
| EU708737 | FP.VHSV.411 | Dau24-02 | 2002 | Germany | *Oncorhynchus mykiss* | 145-Ia-9 |
| EU708741 | FP.VHSV.407 | Dau56-02 | 2002 | Germany | *Oncorhynchus mykiss* | 147-Ia-8 |
| EU708744 | FP.VHSV.404 | Dau412-02 | 2002 | Germany | *Oncorhynchus mykiss* | 149-Ia-9 |
| EU708786 | FP.VHSV.363 | Dsa6915-02 | 2002 | Germany | *Oncorhynchus mykiss* | 179-Ia-8 |
| EU708747 | FP.VHSV.401 | Dau543-03 | 2003 | Germany | *Oncorhynchus mykiss* | 151-Ia-9 |
| EU708758 | FP.VHSV.390 | Db905-03 | 2003 | Germany | *Oncorhynchus mykiss* | 159-Ia-9 |
| EU708771 | FP.VHSV.378 | Dns13462-03 | 2003 | Germany | *Oncorhynchus mykiss* | 166-Ia-9 |
| EU708790 | FP.VHSV.359 | Dsa21583-03 | 2003 | Germany | *Oncorhynchus mykiss* | 182-Ia-8 |
| EU708794 | Dstg22-03 | Dstg22-03 | 2003 | Germany | *Oncorhynchus mykiss* | 10-Ia-5 |
| EU708795 | FP.VHSV.354 | Dstg37-03 | 2003 | Germany | *Oncorhynchus mykiss* | 183-Ia-9 |
| EU708797 | FP.VHSV.352 | Dstg38-03 | 2003 | Germany | *Oncorhynchus mykiss* | 183-Ia-9 |
| EU708799 | FP.VHSV.350 | Dstg49-03 | 2003 | Germany | *Oncorhynchus mykiss* | 183-Ia-9 |
| EU708801 | FP.VHSV.348 | Dstg74-03 | 2003 | Germany | *Oncorhynchus mykiss* | 183-Ia-9 |
| EU708802 | FP.VHSV.347 | Dstg84-03 | 2003 | Germany | *Oncorhynchus mykiss* | 186-Ia-9 |
| EU708813 | FP.VHSV.336 | Dwb48-03 | 2003 | Germany | *Oncorhynchus mykiss* | 186-Ia-9 |
| LN876943 | FP.VHSV.860 | V01-03sa | 2003 | Germany | *Oncorhynchus mykiss* | 226-Ia-8 |
| EU708742 | FP.VHSV.406 | Dau170-04 | 2004 | Germany | *Oncorhynchus mykiss* | 2-Ia-4 |
| EU708796 | FP.VHSV.353 | Dstg37-04 | 2004 | Germany | *Oncorhynchus mykiss* | 184-Ia-9 |
| EU708798 | FP.VHSV.351 | Dstg44-04 | 2004 | Germany | *Oncorhynchus mykiss* | 185-Ia-9 |
| EU708800 | FP.VHSV.349 | Dstg50-2-04 | 2004 | Germany | *Oncorhynchus mykiss* | 184-Ia-9 |
| EU708814 | FP.VHSV.335 | Dwb86-04 | 2004 | Germany | *Oncorhynchus mykiss* | 196-Ia-9 |
| EU708815 | FP.VHSV.334 | Dwb88-04 | 2004 | Germany | *Oncorhynchus mykiss* | 186-Ia-9 |
| EU708816 | FP.VHSV.333 | Dwb97-04 | 2004 | Germany | *Oncorhynchus mykiss* | 186-Ia-9 |
| EU708743 | FP.VHSV.405 | Dau201-05 | 2005 | Germany | *Oncorhynchus mykiss* | 148-Ia-9 |
| EU708760 | FP.VHSV.389 | Dfr195-05 | 2005 | Germany | *Oncorhynchus mykiss* | 4-Ia-3 |
| EU708761 | FP.VHSV.388 | Dfr404-05 | 2005 | Germany | *Oncorhynchus mykiss* | 160-Ia-9 |
| EU708773 | FP.VHSV.376 | Dri07-05 | 2005 | Germany | *Oncorhynchus mykiss* | 167-Ia-8 |
| EU708787 | FP.VHSV.362 | Dsa14893-05 | 2005 | Germany | *Oncorhynchus mykiss* | 180-Ia-8 |
| EU708788 | FP.VHSV.361 | Dsa14894-05 | 2005 | Germany | *Oncorhynchus mykiss* | 181-Ia-9 |
| EU708789 | FP.VHSV.360 | Dsa16257-05 | 2005 | Germany | *Esox lucius* | 180-Ia-8 |
| LN876944 | FP.VHSV.861 | V01-05bb | 2005 | Germany | *Oncorhynchus mykiss* | 124-Ia-5 |
| LN876945 | FP.VHSV.862 | V02-05bb | 2005 | Germany | *Oncorhynchus mykiss* | 40-Ia-5 |
| LN876946 | FP.VHSV.863 | V03-05bb | 2005 | Germany | *Oncorhynchus mykiss* | 40-Ia-5 |
| EU708764 | FP.VHSV.385 | Dfr2247-06 | 2006 | Germany | *Oncorhynchus mykiss* | 163-Ia-9 |
| EU708765 | FP.VHSV.384 | Dfr2868-06 | 2006 | Germany | *Oncorhynchus mykiss* | 164-Ia-9 |
| EU708770 | FP.VHSV.379 | Dns166-06 | 2006 | Germany | *Oncorhynchus mykiss* | 165-Ia-9 |
| EU708772 | FP.VHSV.377 | Dri01-06 | 2006 | Germany | *Thymallus thymallus* | 7-Ia-5 |
| EU708776 | FP.VHSV.373 | Dri47-06 | 2006 | Germany | *Oncorhynchus mykiss* | 169-Ia-8 |
| EU708803 | FP.VHSV.346 | Dstg8-06 | 2006 | Germany | *Oncorhynchus mykiss* | 187-Ia-9 |
| EU708805 | FP.VHSV.344 | Dstg28-06 | 2006 | Germany | *Oncorhynchus mykiss* | 189-Ia-9 |
| EU708807 | FP.VHSV.342 | Dstg36-06 | 2006 | Germany | *Oncorhynchus mykiss* | 191-Ia-9 |
| EU708808 | FP.VHSV.341 | Dstg42-06 | 2006 | Germany | *Oncorhynchus mykiss* | 192-Ia-9 |
| LN876947 | FP.VHSV.864 | V02-06he | 2006 | Germany | *Oncorhynchus mykiss* | 239-Ia-8 |
| EU708762 | FP.VHSV.387 | Dfr148-07 | 2007 | Germany | *Oncorhynchus mykiss* | 161-Ia-9 |
| EU708763 | FP.VHSV.386 | Dfr378-07 | 2007 | Germany | *Oncorhynchus mykiss* | 162-Ia-9 |
| EU708767 | FP.VHSV.382 | Dns6-07 | 2007 | Germany | *Oncorhynchus mykiss* | 5-Ia-5 |
| EU708768 | FP.VHSV.381 | Dns7-07 | 2007 | Germany | *Oncorhynchus mykiss* | 5-Ia-5 |
| EU708769 | FP.VHSV.380 | Dns46-07 | 2007 | Germany | *Oncorhynchus mykiss* | 6-Ia-5 |
| EU708792 | FP.VHSV.357 | Dsh-fv2-07 | 2007 | Germany | *Oncorhynchus mykiss* | 5-Ia-5 |
| EU708804 | FP.VHSV.345 | Dstg21-07 | 2007 | Germany | *Oncorhynchus mykiss* | 188-Ia-9 |
| EU708806 | FP.VHSV.343 | Dstg31-07 | 2007 | Germany | *Oncorhynchus mykiss* | 190-Ia-9 |
| EU708809 | FP.VHSV.340 | Dstg54-1-07 | 2007 | Germany | *Oncorhynchus mykiss* | 193-Ia-9 |
| EU708810 | FP.VHSV.339 | Dstg82-1-07 | 2007 | Germany | *Oncorhynchus mykiss* | 194-Ia-8 |
| EU708811 | FP.VHSV.338 | Dstg89-1-07 | 2007 | Germany | *Oncorhynchus mykiss* | 195-Ia-8 |
| LN876948 | FP.VHSV.865 | D-nwfv185-07 | 2007 | Germany | *Oncorhynchus mykiss* | 397-Ia-8 |
| LN876949 | FP.VHSV.1124 | V02-07sa | 2007 | Germany | *Oncorhynchus mykiss* | 188-Ia-9 |
| EU708757 | FP.VHSV.391 | Db493-08 | 2008 | Germany | *Oncorhynchus mykiss* | 158-Ia-9 |
| EU708775 | FP.VHSV.374 | Dri04-08 | 2008 | Germany | *Oncorhynchus mykiss* | 168-Ia-9 |
| LN876950 | FP.VHSV.866 | D-nsav212-08 | 2008 | Germany | *Oncorhynchus mykiss* | 398-Ia-8 |
| LN876951 | FP.VHSV.867 | V02-08ns | 2008 | Germany | *Oncorhynchus mykiss* | 240-Ia-9; 241-Ia-9 |
| LN876952 | FP.VHSV.868 | V03-08by | 2008 | Germany | *Oncorhynchus mykiss* | 248-Ia-9 |
| LN876953 | FP.VHSV.869 | V04-08by | 2008 | Germany | *Oncorhynchus mykiss* | 251-Ia-9 |
| LN876954 | FP.VHSV.870 | V05-08by | 2008 | Germany | *Oncorhynchus mykiss* | 253-Ia-9 |
| LN876955 | FP.VHSV.871 | V09-08bw | 2008 | Germany | *Oncorhynchus mykiss* | 169-Ia-8 |
| LN876956 | FP.VHSV.872 | V10-08bw | 2008 | Germany | *Oncorhynchus mykiss* | 169-Ia-8 |
| LN876957 | FP.VHSV.873 | V11-08bw | 2008 | Germany | *Oncorhynchus mykiss* | 169-Ia-8 |
| LN876958 | FP.VHSV.874 | V12-08sa | 2008 | Germany | *Oncorhynchus mykiss* | 168-Ia-9 |
| LN876959 | FP.VHSV.875 | V13-08sa | 2008 | Germany | *Oncorhynchus mykiss* | 262-Ia-9 |
| LN876960 | FP.VHSV.876 | V14-08sa | 2008 | Germany | *Oncorhynchus mykiss* | 262-Ia-9 |
| LN876961 | FP.VHSV.877 | V15-08ns | 2008 | Germany | *Oncorhynchus mykiss* | 130-Ia-5 |
| LN876962 | FP.VHSV.878 | V16-08sx | 2008 | Germany | *Oncorhynchus mykiss* | 271-Ia-9 |
| LN876963 | FP.VHSV.879 | V17-08rp | 2008 | Germany | *Oncorhynchus mykiss* | 273-Ia-9 |
| LN876964 | FP.VHSV.880 | V18-08rp | 2008 | Germany | *Oncorhynchus mykiss* | 378-Ia-9 |
| LN876965 | FP.VHSV.881 | V19-08rp | 2008 | Germany | *Oncorhynchus mykiss* | 273-Ia-9 |
| LN876966 | FP.VHSV.882 | V24-08rp | 2008 | Germany | *Oncorhynchus mykiss* | 200-Ia-9 |
| LN876967 | FP.VHSV.883 | V25-08mv | 2008 | Germany | *Salmo trutta* | 267-Ia-9 |
| LN876968 | FP.VHSV.884 | V26-08mv | 2008 | Germany | *Salmo trutta* | 267-Ia-9 |
| LN876969 | FP.VHSV.885 | V27-08mv | 2008 | Germany | *Salmo trutta* | 287-Ia-9 |
| LN876970 | FP.VHSV.886 | V28-08mv | 2008 | Germany | *Esox lucius ** | 290-Ia-9 |
| LN876971 | FP.VHSV.887 | V29-08he | 2008 | Germany | *Oncorhynchus mykiss* | 292-Ia-9 |
| LN876972 | FP.VHSV.888 | V30-08by | 2008 | Germany | *Oncorhynchus mykiss* | 294-Ia-9 |
| LN876973 | FP.VHSV.889 | V31-08by | 2008 | Germany | *Oncorhynchus mykiss* | 298-Ia-9 |
| LN876974 | FP.VHSV.890 | V32-08by | 2008 | Germany | *Oncorhynchus mykiss* | 200-Ia-9 |
| LN876975 | FP.VHSV.891 | V35-08by | 2008 | Germany | *Oncorhynchus mykiss* | 302-Ia-9 |
| LN876976 | FP.VHSV.892 | V36-08by | 2008 | Germany | *Oncorhynchus mykiss* | 303-Ia-9 |
| LN876977 | FP.VHSV.893 | V37-08by | 2008 | Germany | *Oncorhynchus mykiss* | 304-Ia-9; 305-Ia-9 |
| LN876978 | FP.VHSV.894 | V38-08by | 2008 | Germany | *Oncorhynchus mykiss* | 279-Ia-9 |
| LN876979 | FP.VHSV.895 | V39-08by | 2008 | Germany | *Oncorhynchus mykiss* | 307-Ia-9 |
| LN876980 | FP.VHSV.896 | V44-08he | 2008 | Germany | *Oncorhynchus mykiss x Salmon trutta* | 292-Ia-9 |
| LN876981 | FP.VHSV.897 | V01-09ns | 2009 | Germany | *Oncorhynchus mykiss* | 229-Ia-9 |
| LN876982 | FP.VHSV.898 | V02-09ns | 2009 | Germany | *Oncorhynchus mykiss* | 229-Ia-9 |
| LN876983 | FP.VHSV.899 | V03-09ns | 2009 | Germany | *Oncorhynchus mykiss* | 229-Ia-9 |
| LN876984 | FP.VHSV.900 | V04-09ns | 2009 | Germany | *Oncorhynchus mykiss* | 130-Ia-5 |
| LN876985 | FP.VHSV.901 | V05-09ns | 2009 | Germany | *Oncorhynchus mykiss* | 130-Ia-5 |
| LN876986 | FP.VHSV.902 | V06-09ns | 2009 | Germany | *Oncorhynchus mykiss* | 255-Ia-9 |
| LN876987 | FP.VHSV.903 | V08-09sa | 2009 | Germany | *Oncorhynchus mykiss* | 168-Ia-9 |
| LN876988 | FP.VHSV.904 | V09-09bw | 2009 | Germany | *Oncorhynchus mykiss* | 258-Ia-9 |
| LN876989 | FP.VHSV.905 | V10-09bw | 2009 | Germany | *Oncorhynchus mykiss* | 258-Ia-9 |
| LN876990 | FP.VHSV.906 | V11-09sx | 2009 | Germany | *Oncorhynchus mykiss* | 262-Ia-9 |
| LN876991 | FP.VHSV.907 | V12-09sx | 2009 | Germany | *Oncorhynchus mykiss* | 262-Ia-9 |
| LN876992 | FP.VHSV.908 | V13-09sx | 2009 | Germany | *Oncorhynchus mykiss* | 266-Ia-9 |
| LN876993 | FP.VHSV.909 | V14-09sx | 2009 | Germany | *Oncorhynchus mykiss* | 268-Ia-9 |
| LN876994 | FP.VHSV.910 | V15-09sx | 2009 | Germany | *Oncorhynchus mykiss* | 269-Ia-9 |
| LN876995 | FP.VHSV.911 | V16-09sx | 2009 | Germany | *Oncorhynchus mykiss* | 262-Ia-9 |
| LN876996 | FP.VHSV.912 | V17-09sx | 2009 | Germany | *Oncorhynchus mykiss* | 274-Ia-9 |
| LN876997 | FP.VHSV.913 | V18-09sx | 2009 | Germany | *Oncorhynchus mykiss* | 277-Ia-9 |
| LN876998 | FP.VHSV.914 | V23-09rp | 2009 | Germany | *Oncorhynchus mykiss* | 200-Ia-9 |
| LN876999 | FP.VHSV.915 | V24-09rp | 2009 | Germany | *Oncorhynchus mykiss* | 200-Ia-9 |
| LN877000 | FP.VHSV.916 | V25-09rp | 2009 | Germany | *Oncorhynchus mykiss* | 200-Ia-9 |
| LN877001 | FP.VHSV.917 | V27-09rp | 2009 | Germany | *Oncorhynchus mykiss* | 200-Ia-9 |
| LN877002 | FP.VHSV.918 | V28-09rp | 2009 | Germany | *Oncorhynchus mykiss* | 200-Ia-9 |
| LN877003 | FP.VHSV.919 | V30-09mv | 2009 | Germany | *Oncorhynchus mykiss* | 295-Ia-9 |
| LN877004 | FP.VHSV.920 | V31-09mv | 2009 | Germany | *Esox lucius ^*^* | 229-Ia-9 |
| LN877005 | FP.VHSV.921 | V32-09he | 2009 | Germany | *Oncorhynchus mykiss* | 263-Ia-9 |
| LN877006 | FP.VHSV.922 | V33-09th | 2009 | Germany | *Oncorhynchus mykiss* | 300-Ia-9 |
| LN877007 | FP.VHSV.923 | V34-09by | 2009 | Germany | *Oncorhynchus mykiss* | 301-Ia-9 |
| LN877008 | FP.VHSV.924 | V35-09by | 2009 | Germany | *Oncorhynchus mykiss* | 258-Ia-9 |
| LN877009 | FP.VHSV.925 | V36-09by | 2009 | Germany | *Oncorhynchus mykiss* | 258-Ia-9 |
| LN877010 | FP.VHSV.926 | V37-09by | 2009 | Germany | *Oncorhynchus mykiss* | 306-Ia-8 |
| LN877011 | FP.VHSV.927 | V38-09by | 2009 | Germany | *Oncorhynchus mykiss* | 135-Ia-4 |
| LN877012 | FP.VHSV.928 | V40-09he | 2009 | Germany | *Oncorhynchus mykiss* | 200-Ia-9; 310-Ia-9 |
| LN877013 | FP.VHSV.929 | V01-10by | 2010 | Germany | *Oncorhynchus mykiss* | 230-Ia-9 |
| LN877014 | FP.VHSV.930 | V02-10ns | 2010 | Germany | *Oncorhynchus mykiss* | 242-Ia-9 |
| LN877015 | FP.VHSV.931 | V03-10sa | 2010 | Germany | *Oncorhynchus mykiss* | 229-Ia-9 |
| LN877016 | FP.VHSV.932 | V04-10bb | 2010 | Germany | *Oncorhynchus mykiss* | 168-Ia-9 |
| LN877017 | FP.VHSV.933 | V05-10bb | 2010 | Germany | *Oncorhynchus mykiss* | 168-Ia-9 |
| LN877018 | FP.VHSV.934 | V06-10bw | 2010 | Germany | *Oncorhynchus mykiss* | 256-Ia-9 |
| LN877019 | FP.VHSV.935 | V07-10sx | 2010 | Germany | *Oncorhynchus mykiss* | 35-Ia-5 |
| LN877020 | FP.VHSV.936 | V08-10sx | 2010 | Germany | *Oncorhynchus mykiss* | 229-Ia-9 |
| LN877021 | FP.VHSV.937 | V09-10sx | 2010 | Germany | *Oncorhynchus mykiss* | 200-Ia-9 |
| LN877022 | FP.VHSV.938 | V10-10nw | 2010 | Germany | *Lota lota* | 260-Ia-9 |
| LN877023 | FP.VHSV.939 | V11-10sx | 2010 | Germany | *Oncorhynchus mykiss* | 229-Ia-9 |
| LN877024 | FP.VHSV.940 | V12-10mv | 2010 | Germany | *Oncorhynchus mykiss* | 265-Ia-9 |
| LN877025 | FP.VHSV.941 | V13-10mv | 2010 | Germany | *Oncorhynchus mykiss* | 267-Ia-9 |
| LN877026 | FP.VHSV.942 | V14-10bb | 2010 | Germany | *Oncorhynchus mykiss* | 168-Ia-9 |
| LN877027 | FP.VHSV.943 | V20-10sx | 2010 | Germany | *Oncorhynchus mykiss* | 229-Ia-9 |
| LN877028 | FP.VHSV.944 | V21-10bw | 2010 | Germany | *Oncorhynchus mykiss* | 282-Ia-8; 388-Ia-8 |
| LN877029 | FP.VHSV.945 | V22-10bw | 2010 | Germany | *Oncorhynchus mykiss* | 282-Ia-8 |
| LN877030 | FP.VHSV.946 | V23-10bw | 2010 | Germany | *Oncorhynchus mykiss* | 282-Ia-8 |
| LN877031 | FP.VHSV.947 | V24-10bw | 2010 | Germany | *Oncorhynchus mykiss* | 282-Ia-8 |
| LN877032 | FP.VHSV.948 | V25-10bw | 2010 | Germany | *Oncorhynchus mykiss* | 282-Ia-8 |
| LN877033 | FP.VHSV.949 | V26-10sx | 2010 | Germany | *Oncorhynchus mykiss* | 286-Ia-9 |
| LN877034 | FP.VHSV.950 | V27-10bb | 2010 | Germany | *Oncorhynchus mykiss* | 288-Ia-9 |
| LN877035 | FP.VHSV.951 | V30-10by | 2010 | Germany | *Oncorhynchus mykiss* | 296-Ia-9 |
| LN877036 | FP.VHSV.952 | V31-10by | 2010 | Germany | *Salmo trutta fario* | 299-Ia-9 |
| LN877037 | FP.VHSV.953 | V32-10by | 2010 | Germany | *Oncorhynchus mykiss* | 263-Ia-9 |
| LN877038 | FP.VHSV.999 | V01-11bw | 2011 | Germany | *Oncorhynchus mykiss* | 200-Ia-9 |
| LN877039 | FP.VHSV.1000 | V02-11bw | 2011 | Germany | *Oncorhynchus mykiss* | 243-Ia-9 |
| LN877040 | FP.VHSV.1001 | V03-11bw | 2011 | Germany | *Oncorhynchus mykiss* | 243-Ia-9 |
| LN877041 | FP.VHSV.1002 | V04-11bw | 2011 | Germany | *Oncorhynchus mykiss* | 252-Ia-9 |
| LN877042 | FP.VHSV.1003 | V05-11sa | 2011 | Germany | *Oncorhynchus mykiss* | 254-Ia-9 |
| LN877043 | FP.VHSV.1004 | V06-11sa | 2011 | Germany | *Oncorhynchus mykiss* | 254-Ia-9 |
| LN877044 | FP.VHSV.1005 | V07-11sx | 2011 | Germany | *Oncorhynchus mykiss* | 257-Ia-9 |
| LN877045 | FP.VHSV.1006 | V08-11sx | 2011 | Germany | *Oncorhynchus mykiss* | 131-Ia-5 |
| LN877046 | FP.VHSV.1007 | V09-11bw | 2011 | Germany | *Oncorhynchus mykiss* | 259-Ia-9 |
| LN877047 | FP.VHSV.1008 | V10-11bw | 2011 | Germany | *Oncorhynchus mykiss* | 259-Ia-9 |
| LN877048 | FP.VHSV.1009 | V11-11sh | 2011 | Germany | *Coregonus spec.* | 263-Ia-9 |
| LN877049 | FP.VHSV.1010 | V12-11sh | 2011 | Germany | *Coregonus spec.* | 263-Ia-9 |
| LN877050 | FP.VHSV.1011 | V13-11sh | 2011 | Germany | *Percina spec.* | 263-Ia-9 |
| LN877051 | FP.VHSV.1012 | V14-11sx | 2011 | Germany | *Oncorhynchus mykiss* | 264-Ia-9 |
| LN877052 | FP.VHSV.1013 | V17-11by | 2011 | Germany | *Oncorhynchus mykiss* | 267-Ia-9 |
| LN877053 | FP.VHSV.1014 | V18-11by | 2011 | Germany | *Oncorhynchus mykiss* | 279-Ia-9 |
| LN877054 | FP.VHSV.1015 | V19-11by | 2011 | Germany | *Oncorhynchus mykiss* | 281-Ia-8 |
| LN877055 | FP.VHSV.1016 | V20-11by | 2011 | Germany | *Oncorhynchus mykiss* | 133-Ia-4; 134-Ia-4 |
| LN877056 | FP.VHSV.1017 | V21-11rp | 2011 | Germany | *Salmo trutta* | 200-Ia-9 |
| LN877057 | FP.VHSV.1018 | V22-11rp | 2011 | Germany | *Salmo trutta* | 200-Ia-9 |
| LN877058 | FP.VHSV.1019 | V23-11rp | 2011 | Germany | *Salmo trutta* | 200-Ia-9 |
| LN877059 | FP.VHSV.1020 | V25-11rp | 2011 | Germany | *Salmo trutta* | 221-Ia-9 |
| LN877060 | FP.VHSV.1021 | V26-11rp | 2011 | Germany | *Salmo trutta* | 221-Ia-9 |
| LN877061 | FP.VHSV.1022 | V01-12sx | 2012 | Germany | unknown | 231-Ia-9 |
| LN877062 | FP.VHSV.1023 | V02-12ns | 2012 | Germany | *Oncorhynchus mykiss* | 244-Ia-9 |
| LN877063 | FP.VHSV.1024 | V03-12ns | 2012 | Germany | *Oncorhynchus mykiss* | 244-Ia-9 |
| LN877064 | FP.VHSV.1025 | V04-12ns | 2012 | Germany | *Oncorhynchus mykiss* | 244-Ia-9 |
| LN877065 | FP.VHSV.1026 | V05-12ns | 2012 | Germany | *Oncorhynchus mykiss* | 244-Ia-9 |
| LN877066 | FP.VHSV.1027 | V06-12ns | 2012 | Germany | *Oncorhynchus mykiss* | 244-Ia-9 |
| LN877067 | FP.VHSV.1028 | V07-12ns | 2012 | Germany | *Oncorhynchus mykiss* | 244-Ia-9 |
| LN877068 | FP.VHSV.1029 | V10-12sa | 2012 | Germany | *Oncorhynchus mykiss* | 261-Ia-8 |
| LN877069 | FP.VHSV.1030 | V11-12sx | 2012 | Germany | *Oncorhynchus mykiss* | 264-Ia-9 |
| LN877070 | FP.VHSV.1031 | V12-12sx | 2012 | Germany | *Oncorhynchus mykiss* | 264-Ia-9 |
| LN877071 | FP.VHSV.1032 | V13-12sx | 2012 | Germany | *Oncorhynchus mykiss* | 264-Ia-9 |
| LN877072 | FP.VHSV.1033 | V14-12sa | 2012 | Germany | *Oncorhynchus mykiss* | 132-Ia-4 |
| LN877073 | FP.VHSV.1034 | V15-12sa | 2012 | Germany | *Oncorhynchus mykiss* | 132-Ia-4 |
| LN877074 | FP.VHSV.1035 | V16-12bw | 2012 | Germany | *Salmo trutta* | 272-Ia-8 |
| LN877075 | FP.VHSV.1036 | V17-12nw | 2012 | Germany | *Oncorhynchus mykiss* | 276-Ia-8 |
| LN877076 | FP.VHSV.1037 | V18-12nw | 2012 | Germany | *Oncorhynchus mykiss* | 280-Ia-8 |
| LN877077 | FP.VHSV.1037 | V19-12nw | 2012 | Germany | *Oncorhynchus mykiss* | 276-Ia-8 |
| LN877078 | FP.VHSV.1039 | V20-12nw | 2012 | Germany | *Salmo trutta* | 276-Ia-8 |
| LN877079 | FP.VHSV.1040 | V21-12nw | 2012 | Germany | *Oncorhynchus mykiss* | 276-Ia-8 |
| LN877080 | FP.VHSV.1041 | V22-12nw | 2012 | Germany | *Oncorhynchus mykiss* | 276-Ia-8 |
| LN877081 | FP.VHSV.1042 | V23-12by | 2012 | Germany | *Oncorhynchus mykiss* | 283-Ia-8; 284-Ia-8 |
| LN877082 | FP.VHSV.1043 | V24-12bw | 2012 | Germany | *Oncorhynchus mykiss,Salvelinus spec.* | 285-Ia-8 |
| LN877083 | FP.VHSV.1044 | V25-12bw | 2012 | Germany | *Oncorhynchus mykiss,Salvelinus spec.* | 285-Ia-8 |
| LN877084 | FP.VHSV.1045 | V26-12bw | 2012 | Germany | *Oncorhynchus mykiss,Salvelinus spec.* | 285-Ia-8 |
| LN877085 | FP.VHSV.1046 | V27-12by | 2012 | Germany | *Oncorhynchus mykiss* | 289-Ia-9 |
| LN877086 | FP.VHSV.1047 | V28-12by | 2012 | Germany | *Oncorhynchus mykiss* | 291-Ia-8 |
| LN877087 | FP.VHSV.1048 | V29-12by | 2012 | Germany | *Oncorhynchus mykiss* | 293-Ia-8 |
| LN877088 | FP.VHSV.1049 | V30-12ns | 2012 | Germany | *Salmo trutta* | 297-Ia-8 |
| LN877089 | FP.VHSV.1050 | V31-12by | 2012 | Germany | *Oncorhynchus mykiss* | 385-Ia-4 |
| LN877090 | FP.VHSV.1051 | V32-12rp | 2012 | Germany | *Salmo trutta* | 221-Ia-9 |
| LN877091 | FP.VHSV.1052 | V33-12sx | 2012 | Germany | *Oncorhynchus mykiss* | 229-Ia-9 |
| LN877092 | FP.VHSV.954 | V01-13by | 2013 | Germany | *Oncorhynchus mykiss* | 361-Ia-8 |
| LN877093 | \| FP.VHSV.955 | V02-13by | 2013 | Germany | *Oncorhynchus mykiss* | 362-Ia-4 |
| LN877094 | FP.VHSV.956 | V03-13bw | 2013 | Germany | *Oncorhynchus mykiss* | 361-Ia-8 |
| LN877095 | FP.VHSV.957 | V04-13bw | 2013 | Germany | *Salmonidae* | 361-Ia-8 |
| LN877096 | FP.VHSV.958 | V05-13bw | 2013 | Germany | *Salmonidae* | 361-Ia-8 |
| LN877097 | FP.VHSV.959 | V08-13by | 2013 | Germany | *Oncorhynchus mykiss* | 360-Ia-8 |
| LN877098 | FP.VHSV.960 | V09-13by | 2013 | Germany | *Salmo trutta* and other *Salmonidae* | 359-Ia-8 |
| LN877099 | FP.VHSV.961 | V10-13bw | 2013 | Germany | *Oncorhynchus mykiss* | 205-Ia-8 |
| LN877100 | FP.VHSV.962 | V11-13bw | 2013 | Germany | *Oncorhynchus mykiss* | 205-Ia-8 |
| LN877101 | FP.VHSV.963 | V12-13sx | 2013 | Germany | *Oncorhynchus mykiss* | 358-Ia-8 |
| LN877102 | FP.VHSV.964 | V13-13bw | 2013 | Germany | *Oncorhynchus mykiss* | 357-Ia-9 |
| LN877103 | FP.VHSV.965 | V14-13bw | 2013 | Germany | *Oncorhynchus mykiss* | 387-Ia-9 |
| LN877104 | FP.VHSV.966 | V16-13by | 2013 | Germany | *Salmo trutta fario* | 392-Ia-9; 393-Ia-9 |
| LN877105 | FP.VHSV.967 | V01-14bb | 2014 | Germany | *Salmo trutta* | 229-Ia-9 |
| LN877106 | FP.VHSV.968 | V02-14sx | 2014 | Germany | *Oncorhynchus mykiss* | 348-Ia-8 |
| LN877107 | FP.VHSV.969 | V03-14bb | 2014 | Germany | *Oncorhynchus mykiss* | 349-Ia-8 |
| LN877108 | FP.VHSV.970 | V04-14nw | 2014 | Germany | *Oncorhynchus mykiss* | 350-Ia-8 |
| LN877109 | FP.VHSV.971 | V05-14nw | 2014 | Germany | *Oncorhynchus mykiss* | 350-Ia-8 |
| LN877110 | FP.VHSV.972 | V06-14sa | 2014 | Germany | *Oncorhynchus mykiss* | 348-Ia-8 |
| LN877111 | FP.VHSV.973 | V07-14bw | 2014 | Germany | *Oncorhynchus mykiss* | 351-Ia-8 |
| LN877112 | FP.VHSV.974 | V08-14bw | 2014 | Germany | *Oncorhynchus mykiss* | 351-Ia-8 |
| LN877113 | FP.VHSV.975 | V09-14bw | 2014 | Germany | *Oncorhynchus mykiss* | 351-Ia-8; 363-Ia-8 |
| LN877114 | FP.VHSV.976 | V29-14sx | 2014 | Germany | *Oncorhynchus mykiss* | 352-Ia-8 |
| LN877115 | FP.VHSV.977 | V30-14bw | 2014 | Germany | *Oncorhynchus mykiss* | 353-Ia-8 |
| LN877116 | FP.VHSV.978 | V31-14bw | 2014 | Germany | *Oncorhynchus mykiss* | 353-Ia-8 |
| LN877117 | FP.VHSV.979 | V38-14nw | 2014 | Germany | *Oncorhynchus mykiss* | 354-Ia-8 |
| LN877118 | FP.VHSV.980 | V39-14by | 2014 | Germany | *Oncorhynchus mykiss* | 355-Ia-9 |
| LN877119 | FP.VHSV.981 | V40-14by | 2014 | Germany | *Oncorhynchus mykiss* | 355-Ia-9 |
| LN877120 | FP.VHSV.982 | V41-14bb | 2014 | Germany | *Salmo trutta* | 356-Ia-8 |
| LN877121 | FP.VHSV.983 | V43-14bb | 2014 | Germany | *Oncorhynchus mykiss* | 356-Ia-8 |
| LN877122 | FP.VHSV.984 | V44-14bb | 2014 | Germany | *Oncorhynchus mykiss* | 356-Ia-8 |
| LN877123 | FP.VHSV.985 | V45-14bb | 2014 | Germany | *Oncorhynchus mykiss* | 371-Ia-8 |
| LN877124 | FP.VHSV.986 | V50-14by | 2014 | Germany | *Oncorhynchus mykiss* | 370-Ia-8 |
| LN877125 | FP.VHSV.987 | V51-14sx | 2014 | Germany | *Oncorhynchus mykiss* | 369-Ia-8 |
| LN877126 | FP.VHSV.988 | V52-14sx | 2014 | Germany | *Oncorhynchus mykiss* | 369-Ia-8 |
| LN877127 | FP.VHSV.989 | V53-14bw | 2014 | Germany | *Oncorhynchus mykiss* | 368-Ia-9 |
| LN877128 | FP.VHSV.990 | V54-14bw | 2014 | Germany | *Oncorhynchus mykiss* | 367-Ia-9 |
| LN877129 | FP.VHSV.991 | V55-14bw | 2014 | Germany | *Oncorhynchus mykiss* | 366-Ia-8 |
| LN877130 | FP.VHSV.992 | V56-14bw | 2014 | Germany | *Oncorhynchus mykiss* | 366-Ia-8 |
| LN877131 | FP.VHSV.993 | V57-14bw | 2014 | Germany | *Oncorhynchus mykiss* | 365-Ia-8 |
| LN877132 | FP.VHSV.994 | V58-14by | 2014 | Germany | *Oncorhynchus mykiss* | 396-Ia-8 |
| LN877133 | FP.VHSV.995 | V60-14by | 2014 | Germany | *Oncorhynchus mykiss* | 355-Ia-9; 395-Ia-9 |
| LN877134 | FP.VHSV.996 | V61-14sa | 2014 | Germany | *Oncorhynchus mykiss* | 390-Ia-8 |
| LN877135 | FP.VHSV.997 | V02-15by | 2015 | Germany | *Oncorhynchus mykiss* | 355-Ia-9 |
| LN877136 | FP.VHSV.998 | V03-15sa | 2015 | Germany | *Oncorhynchus mykiss* | 391-Ia-8 |
| AJ233396 | FP.VHSV.248 | FR-07-71 | 1971 | France | *Oncorhynchus mykiss* | 137-Ia-6 |
| AY546616 | FP.VHSV.248 | FR-0771 | 1971 | France | *Oncorhynchus mykiss* | 140-Ia-6 |
| AY546617 | FP.VHSV.306 | FR-2375 | 1975 | France | *Oncorhynchus mykiss* | 326-Ia-1 |
| U28799 | FP.VHSV.306 | FR-23-75 | 1975 | France | *Salmo trutta* | 329-Ia-1 |
| U28800 | FP.VHSV.247 | FR-02-84 | 1984 | France | *Salmo trutta* | 324-Ia-**X** |
| AF143863 | FP.VHSV.252 | FR-14-58 | 1990 | France | *Oncorhynchus mykiss* | 325-Ia-1 |
| LN877137 | FP.VHSV.320 | FR-G2192 | 1997 | France | unknown | 198-Ia-3 |
| LN877138 | FP.VHSV.496 | FR-N11298 | 2003 | France | *Esox lucius* | 201-Ia-9 |
| LN877139 | FP.VHSV.321 | FR-N14165-1 | 2004 | France | *Esox lucius* | 202-Ia-8 |
| LN877140 | FP.VHSV.318 | FR-R5509-2 | 2005 | France | *Oncorhynchus mykiss* | 180-Ia-8 |
| LN877141 | FP.VHSV.497 | FR-R374 | 2005 | France | *Oncorhynchus mykiss* | 203-Ia-8 |
| LN877142 | FP.VHSV.317 | FR-FF57 | 2007 | France | *Oncorhynchus mykiss* | 161-Ia-9 |
| LN877143 | FP.VHSV.494 | FR-004439 | 2007 | France | *Oncorhynchus mykiss* | 197-Ia-9 |
| LN877144 | FP.VHSV.316 | FR-GG57 | 2008 | France | *Oncorhynchus mykiss* | 199-Ia-9 |
| LN877145 | FP.VHSV.495 | FR-680401 | 2009 | France | *Oncorhynchus mykiss* | 12-Ia-4 |
| LN877146 | FP.VHSV.498 | FR-JJ40 | 2011 | France | *Oncorhynchus mykiss* | 200-Ia-9 |
| LN877147 | FP.VHSV.319 | FR-1565 | 2012 | France | unknown | 11-Ia-4 |
| EU708732 | FP.VHSV.416 | Au299-94 | 1994 | Austria | *Oncorhynchus mykiss* | 143-Ia-9 |
| AY546570 | FP.VHSV.245 | AU8/95 | 1995 | Austria | *Oncorhynchus mykiss* | 138-Ia-8 |
| EU708729 | FP.VHSV.419 | Au28-95 | 1995 | Austria | *Oncorhynchus mykiss* | 141-Ia-8 |
| EU708731 | FP.VHSV.417 | Au77-99 | 1999 | Austria | *Oncorhynchus mykiss* | 142-Ia-9 |
| EU708733 | FP.VHSV.415 | Au917-04 | 2004 | Austria | *Oncorhynchus mykiss* | 144-Ia-9 |
| LN877148 | FP.VHSV.1053 | V15-13AU | 2013 | Austria | *Oncorhynchus mykiss* | 361-Ia-8 |
| LN877149 | FP.VHSV.1054 | V59-14AU | 2014 | Austria | *Salmo trutta* | 394-Ia-9 |
| AY546571 | FP.VHSV.274 | CH-FI262BFH | 1999 | Switzerland | *Oncorhynchus mykiss* | 139-Ia-9 |
| LN877150 | FP.VHSV.1056 | CH-VE177-00 | 2000 | Switzerland | *Oncorhynchus mykiss* | 222-Ia-9 |
| LN877151 | FP.VHSV.1057 | CH-F144-00 | 2000 | Switzerland | *Oncorhynchus mykiss* | 237-Ia-9 |
| LN877152 | FP.VHSV.1058 | CH-F83-00 | 2000 | Switzerland | *Esox Lucius* | 247-Ia-9 |
| LN877153 | FP.VHSV.1059 | CH-F33-00 | 2000 | Switzerland | *Oncorhynchus mykiss* | 139-Ia-9 |
| LN877154 | FP.VHSV.1060 | CH-F264-01 | 2001 | Switzerland | *Oncorhynchus mykiss* | 223-Ia-9 |
| LN877155 | FP.VHSV.1061 | CH-VE300-1-01 | 2001 | Switzerland | *Oncorhynchus mykiss* | 238-Ia-9 |
| LN877156 | FP.VHSV.1062 | CH-F159-01 | 2001 | Switzerland | *Oncorhynchus mykiss* | 150-Ia-9 |
| LN877157 | FP.VHSV.1063 | CH-VE241-01 | 2001 | Switzerland | *Oncorhynchus mykiss* | 250-Ia-9 |
| LN877158 | FP.VHSV.1064 | CH-F106-02 | 2002 | Switzerland | *Oncorhynchus mykiss* | 224-Ia-9; 225-Ia-9 |
| LN877159 | FP.VHSV.1065 | CH-F245-03 | 2003 | Switzerland | *Oncorhynchus mykiss* | 373-Ia-9 |
| LN877160 | FP.VHSV.1066 | CH-F95-04 | 2004 | Switzerland | *Oncorhynchus mykiss* | 227-Ia-9 |
| LN877161 | FP.VHSV.1067 | CH-F159-06 | 2006 | Switzerland | *Oncorhynchus mykiss* | 228-Ia-9 |
| LN877162 | FP.VHSV.1068 | CH-F259-07 | 2007 | Switzerland | *Oncorhynchus mykiss* | 161-Ia-9 |
| LN877163 | FP.VHSV.1069 | CH-F128-08 | 2008 | Switzerland | *Oncorhynchus mykiss* | 309-Ia-9 |
| LN877164 | FP.VHSV.1070 | CH-F82-08 | 2008 | Switzerland | *Oncorhynchus mykiss* | 311-Ia-9 |
| LN877165 | FP.VHSV.1071 | CH-F56-08 | 2008 | Switzerland | *Oncorhynchus mykiss* | 311-Ia-9 |
| LN877166 | FP.VHSV.1072 | CH-F37-08 | 2008 | Switzerland | *Oncorhynchus mykiss* | 161-Ia-9 |
| LN877167 | FP.VHSV.1073 | CH-F127-08 | 2008 | Switzerland | *Oncorhynchus mykiss* | 309-Ia-9 |
| LN877168 | FP.VHSV.1074 | CH-F142-09 | 2009 | Switzerland | *Oncorhynchus mykiss* | 308-Ia-9 |
| LN877169 | FP.VHSV.1075 | CH-F113-09 | 2009 | Switzerland | *Oncorhynchus mykiss* | 308-Ia-9 |
| LN877170 | FP.VHSV.1076 | CH-F141-09 | 2009 | Switzerland | *Oncorhynchus mykiss* | 308-Ia-9 |
| LN877171 | FP.VHSV.1077 | CH-F177-10 | 2010 | Switzerland | *Oncorhynchus mykiss* | 270-Ia-9 |
| LN877172 | FP.VHSV.1078 | CH-F136-10 | 2010 | Switzerland | *Oncorhynchus mykiss* | 270-Ia-9 |
| LN877173 | FP.VHSV.1079 | CH-F75-10 | 2010 | Switzerland | *Oncorhynchus mykiss* | 275-Ia-9 |
| LN877174 | FP.VHSV.1080 | CH-F69-10 | 2010 | Switzerland | *Oncorhynchus mykiss* | 278-Ia-8 |
| LN877175 | FP.VHSV.1081 | CH-F78E-10 | 2010 | Switzerland | *Oncorhynchus mykiss* | 261-Ia-8 |
| LN877176 | FP.VHSV.1082 | CH-F78B-10 | 2010 | Switzerland | *Oncorhynchus mykiss* | 261-Ia-8 |
| LN877177 | \| FP.VHSV.1083 | CH-F49-10 | 2010 | Switzerland | *Oncorhynchus mykiss* | 261-Ia-8 |
| LN877178 | FP.VHSV.1084 | CH-F70-10 | 2010 | Switzerland | *Oncorhynchus mykiss* | 374-Ia-8 |
| LN877179 | FP.VHSV.1085 | CH-F120-10 | 2010 | Switzerland | *Oncorhynchus mykiss* | 270-Ia-9 |
| LN877180 | FP.VHSV.1086 | CH-F146-12 | 2012 | Switzerland | *Oncorhynchus mykiss* | 375-Ia-9 |
| LN877181 | FP.VHSV.1087 | CH-F340-12 | 2012 | Switzerland | *Oncorhynchus mykiss* | 376-Ia-8 |
| LN877182 | FP.VHSV.1088 | CH-F602-12 | 2012 | Switzerland | *Oncorhynchus mykiss* | 377-Ia-9 |
| LN877183 | FP.VHSV.1089 | CH-F605-12 | 2012 | Switzerland | *Oncorhynchus mykiss* | 377-Ia-9 |
| LN877184 | FP.VHSV.1090 | CH-F607-12 | 2012 | Switzerland | *Oncorhynchus mykiss* | 377-Ia-9 |
| LN877185 | FP.VHSV.1091 | CH-F46-14 | 2014 | Switzerland | *Oncorhynchus mykiss* | 372-Ia-8 |
| JF781264 | FP.VHSV.437 | PL-05/1433 | 2005 | Poland | *Oncorhynchus mykiss* | 322-Ia-8 |
| JF781265 | FP.VHSV.436 | PL-05/5300 | 2005 | Poland | *Oncorhynchus mykiss* | 323-Ia-8 |
| JF781259 | FP.VHSV.443 | PL-06/1247 | 2006 | Poland | *Oncorhynchus mykiss* | 320-Ia-9 |
| JF781260 | FP.VHSV.444 | PL-06/1564 | 2006 | Poland | *Oncorhynchus mykiss* | 321-Ia-9 |
| JF781261 | FP.VHSV.438 | PL-06/5705 | 2006 | Poland | *Oncorhynchus mykiss* | 180-Ia-8 |
| JF781262 | FP.VHSV.439 | PL-06/6260 | 2006 | Poland | *Salvelinus fontinalis* | 180-Ia-8 |
| JF781263 | FP.VHSV.440 | PL-06/9007 | 2006 | Poland | *Oncorhynchus mykiss* | 180-Ia-8 |
| JF781266 | FP.VHSV.442 | PL-06/6258 | 2006 | Poland | *Oncorhynchus mykiss* | 180-Ia-8 |
| JF781267 | FP.VHSV.441 | PL-06/6257 | 2006 | Poland | *Oncorhynchus mykiss* | 180-Ia-8 |
| JF781244 | FP.VHSV.448 | PL-07/1996 | 2007 | Poland | *Oncorhynchus mykiss* | 161-Ia-9 |
| JF781245 | FP.VHSV.445 | PL-07/5055 | 2007 | Poland | *Oncorhynchus mykiss* | 161-Ia-9 |
| JF781247 | FP.VHSV.450 | PL-07/9453 | 2007 | Poland | *Oncorhynchus mykiss* | 161-Ia-9 |
| JF781248 | FP.VHSV.447 | PL-07/1351 | 2007 | Poland | *Oncorhynchus mykiss* | 313-Ia-9 |
| JF781250 | FP.VHSV.449 | PL-07/4007 | 2007 | Poland | *Oncorhynchus mykiss* | 267-Ia-9 |
| JF781258 | FP.VHSV.446 | PL-07/1256 | 2007 | Poland | *Oncorhynchus mykiss* | 319-Ia-9 |
| JF781251 | FP.VHSV.451 | PL-08/2881 | 2008 | Poland | *Oncorhynchus mykiss* | 267-Ia-9 |
| JF781256 | FP.VHSV.452 | PL-08/5429 | 2008 | Poland | *Oncorhynchus mykiss* | 229-Ia-9 |
| JF781257 | FP.VHSV.453 | PL-08/5430 | 2008 | Poland | *Oncorhynchus mykiss* | 229-Ia-9 |
| JF781246 | FP.VHSV.454 | PL-09/3764 | 2009 | Poland | *Oncorhynchus mykiss* | 312-Ia-9 |
| JF781249 | FP.VHSV.455 | PL-09/4330 | 2009 | Poland | *Oncorhynchus mykiss* | 314-Ia-9 |
| JF781252 | FP.VHSV.460 | PL-09/1707-4 | 2009 | Poland | *Oncorhynchus mykiss* | 315-Ia-9 |
| JF781253 | FP.VHSV.433 | PL-09/1708-4 | 2009 | Poland | *Oncorhynchus mykiss* | 315-Ia-9 |
| JF781254 | FP.VHSV.459 | PL-09/1592 | 2009 | Poland | *Oncorhynchus mykiss* | 316-Ia-9 |
| JF781255 | FP.VHSV.456 | PL-09/4332 | 2009 | Poland | *Oncorhynchus mykiss* | 317-Ia-9; 318-Ia-9 |
| GQ292534 | FP.VHSV.308 | 1455/07; SLO 3 | 2007 | Slovenia | *Oncorhynchus mykiss* | 204-Ia-9 |
| LN877186 | FP.VHSV.1092 | VHSV/T.thymallus/I/TN/9/Jan93 | 1993 | Italy | *Thymallus thymallus* | 330-Ia-8 |
| LN877187 | FP.VHSV.1093 | VHSV/O.mykiss/I/PN/688/Dic95 | 1995 | Italy | *Oncorhynchus mykiss* | 331-Ia-9 |
| LN877188 | FP.VHSV.1094 | VHSV/O.mykiss/I/UD/667/Dic96 | 1996 | Italy | *Oncorhynchus mykiss* | 332-Ia-9 |
| LN877189 | FP.VHSV.1095 | VHSV/O.mykiss/I/PN/180/Mar97 | 1997 | Italy | *Oncorhynchus mykiss* | 247-Ia-9 |
| LN877190 | FP.VHSV.1096 | VHSV/O.mykiss/I/TV/604/Nov97 | 1997 | Italy | *Oncorhynchus mykiss* | 333-Ia-9 |
| LN877191 | FP.VHSV.1097 | VHSV/O.mykiss/I/TN/70/Feb00 | 2000 | Italy | *Oncorhynchus mykiss* | 334-Ia-9 |
| LN877192 | FP.VHSV.1098 | VHSV/O.mykiss/I/TN/464/Nov02 | 2002 | Italy | *Oncorhynchus mykiss* | 183-Ia-9 |
| LN877193 | FP.VHSV.1099 | VHSV/O.mykiss/I/VR/81/Mar02 | 2002 | Italy | *Oncorhynchus mykiss* | 335-Ia-9 |
| LN877194 | FP.VHSV.1100 | VHSV/O.mykiss/I/TV/27/Jan03 | 2003 | Italy | *Oncorhynchus mykiss* | 336-Ia-9 |
| LN877195 | FP.VHSV.1101 | VHSV/E.lucius/I/UD/505/Nov03 | 2003 | Italy | *Esox lucius* | 337-Ia-9 |
| LN877196 | FP.VHSV.1102 | VHSV/O.mykiss/I/TV/99/Mar04 | 2004 | Italy | *Oncorhynchus mykiss* | 338-Ia-9 |
| LN877197 | FP.VHSV.1103 | VHSV/O.mykiss/I/VI/49/Feb05 | 2005 | Italy | *Oncorhynchus mykiss* | 339-Ia-9 |
| LN877198 | FP.VHSV.1104 | VHSV/O.mykiss/I/BL/552/Oct05 | 2005 | Italy | *Oncorhynchus mykiss* | 340-Ia-9; 341-Ia-9 |
| LN877199 | FP.VHSV.1105 | VHSV/O.mykiss/I/TN/199/May06 | 2006 | Italy | *Oncorhynchus mykiss* | 342-Ia-9 |
| LN877200 | FP.VHSV.1106 | VHSV/O.mykiss/I/TV/225/Jun07 | 2007 | Italy | *Oncorhynchus mykiss* | 343-Ia-9 |
| LN877201 | FP.VHSV.1107 | VHSV/O.mykiss/I/TN/182/Apr08 | 2008 | Italy | *Oncorhynchus mykiss* | 344-Ia-9 |
| LN877202 | FP.VHSV.1108 | VHSV/O.mykiss/I/TV/251/Jul09 | 2009 | Italy | *Oncorhynchus mykiss* | 345-Ia-9 |
| LN877203 | FP.VHSV.1109 | VHSV/O.mykiss/I/TN/232/May10 | 2010 | Italy | *Oncorhynchus mykiss* | 346-Ia-9 |
| LN877204 | FP.VHSV.1110 | VHSV/O.mykiss/I/BG/332/Sep11 | 2011 | Italy | *Oncorhynchus mykiss* | 262-Ia-9 |
| LN877205 | FP.VHSV.1111 | VHSV/O.mykiss/I/SA/482/Nov11 | 2011 | Italy | *Oncorhynchus mykiss* | 347-Ia-8 |
| AY546621 | FP.VHSV.249 | NO-A16368G | 1968 | Norway | *Oncorhynchus mykiss* | 16-Id |
| LN877206 | FP.VHSV.1116 | DK-3921 | 1987 | Kattegat | *Pleuronectes platessa** | 15-Ib |
| AF143862 | FP.VHSV.253 | 96-43 | 1996 | English Channel | *Clupea harengus** | 1-Ib |
| AY546580 | FP.VHSV.62 | 4p37 | 1997 | North Sea | *Micromesistius poutassou** | 4-Ib |
| FJ460590 | FP.VHSV.62 | 4p37 | 1997 | North Sea | *Micromesistius poutassou** | 5-Ib |
| AY546583 | FP.VHSV.69 | DK-5e59 | 1998 | Kattegat | *Limanda limanda** | 6-Ib |
| AY546622 | FP.VHSV.228 | SE980422217 ^3)^ | 1998 | Sweden | *Oncorhynchus mykiss* | 8-Ib |
| AY546632 | FP.VHSV.213 | UK-MLA98/6PT11 | 1998 | North Sea | *Trisopterus esmarkii** | III |
| AY546584 | FP.VHSV.137 | 6p403 | 1999 | Skagerrak | *Clupea harengus** | 7-Ib |
| EU708766 | FP.VHSV383 | Dglasaal | 1999 | Germany | *Anguilla anguilla* | 12-Ib |
| AY546623, FJ460591 | FP.VHSV.229 | SE000331098^3)^ | 2000 | Sweden | *Oncorhynchus mykiss* | 9-Ib |
| AY546624 | FP.VHSV.329 | SE-010516181-29 | 2000 | Kattegat | *Clupea harengus** | 10-Ib |
| AY546625 | FP.VHSV.330 | SE-010516181-30 | 2000 | Kattegat | *Mallotus villosus*; Sprattus sprattustus** | 11-Ib |
| AY546626 | FP.VHSV.331 | SE-010516181-31 | 2000 | Kattegat | *Clupea harengus** | 10-Ib |
| AY546627 | FP.VHSV.332 | SE-010516181-32 | 2000 | Kattegat | *Pholis gunellus*; Gobiidae*; Zoarces viviparous*; Acanthocottus Scorpius*; poll of bottom-living fish species* | 11-Ib |
| AB839747 | FP.VHSV.1117 | SE-SVA-1033-9C | 2000 | Kattegat | *Oncorhynchus mykiss* | 19-Ib |
| AB839748 | FP.VHSV.1118 | SE-SVA-1033-3F | 2000 | Kattegat | *Oncorhynchus mykiss* | 20-Ib |
| FJ384761 | FP.VHSV.420 | CH150208 | 2008 | North Sea | *Clupea harengus** | 13-Ib |
| JF681360 ^2)^ | FP.VHSV.553 | 2008-50-136-2 | 2008 | Baltic Sea | *Scophthalmus maximus** | 14-Ib |
| HM632035 | FP.VHSV.463 | NO-F/2009 | 2009 | Barents Sea | *Clupea harengus** | 14-Ib |
| KJ768664 | FP.VHSV.1119 | NO-F-GA/2009 | 2009 | Norwegian Sea | *Gadiculus argenteus** | 17-Ib |
| JQ755265 | FP.VHSV.1120 | NO-R/220410-239 | 2010 | Rogaland | *Clupea harengus** | 18-Ib |
| JQ755260 | FP.VHSV.1121 | NO-R/190310-174 | 2010 | Kattegat | *Clupea harengus** | 14-Ib |
| Z93414 | FP.VHSV.36 | M.Rhabdo ^3)^ | 1979 | Baltic Sea | *Gadus morhua ^*^* | 16-Ib |
| AY546572 | FP.VHSV.40 | DK-e62 | 1996 | Baltic Sea | *Gadus morhua ^*^* | 2-Ib |
| AY546573 | FP.VHSV.52 | 1p8 | 1996 | Baltic Sea | *Clupea harengus^*^* | 2-Ib |
| AY546574 | FP.VHSV.51 | 1p12 | 1996 | Baltic Sea | *Clupea harengus^*^* | 2-Ib |
| AY546575 | FP.VHSV.53 | 1p40 | 1996 | Baltic Sea | *Enchelyopus cimbrius^*^* | 3-Ib |
| AY546579 | FP.VHSV.38 | 1p86 | 1996 | Baltic Sea | *Sprattus sprattus^*^* | 4-Ib |
| AY546576 | FP.VHSV.56 | 1p52 | 1996 | Baltic Sea | *Sprattus sprattus^*^* | II |
| AM086354/ AY546614 | FP.VHSV.230 | FiA01a_b.00 / FI-ka66 | 2000 | Baltic Sea/Finland | *Oncorhynchus mykiss* | 1-Id |
| AM086355 | FP.VHSV.843 | FiPO1a_b_c.00 | 2000 | Baltic Sea/Finland | *Oncorhynchus mykiss* | 2-Id |
| AM086356 | FP.VHSV.232 | FiP02a_b.00 | 2000 | Baltic Sea/Finland | *Oncorhynchus mykiss* | 3-Id |
| AY546615 | FP.VHSV.231 | FiP02a.00/ FI-ka422 | 2000 | Baltic Sea/Finland | *Oncorhynchus mykiss* | 3-Id |
| AM086357 | FP.VHSV.1200 | FiP03.00 | 2000 | Baltic Sea/Finland | *Oncorhynchus mykiss* | 4-Id |
| AM086359 | FP.VHSV.1201 | FiA02b.01 | 2001 | Baltic Sea/Finland | *Oncorhynchus mykiss* | 1-Id |
| AM086361 | FP.VHSV.818 | FiA04.01 | 2001 | Baltic Sea/Finland | *Oncorhynchus mykiss* | 1-Id |
| AM086364 | FP.VHSV.819 | FiP03.01 | 2001 | Baltic Sea/Finland | *Oncorhynchus mykiss* | 4-Id |
| AM086358 | FP.VHSV.820 | FiA02a.01 | 2001 | Baltic Sea/Finland | *Oncorhynchus mykiss* | 5-Id |
| AM086360 | FP.VHSV.821 | FiA03a_b.01 | 2001 | Baltic Sea/Finland | *Oncorhynchus mykiss* | 5-Id |
| AM086362 | FP.VHSV.822 | FiA05.01 | 2001 | Baltic Sea/Finland | *Oncorhynchus mykiss* | 6-Id |
| AM086363 | FP.VHSV.823 | FiP04.01 | 2001 | Baltic Sea/Finland | *Oncorhynchus mykiss* | 7-Id |
| AM086365 | FP.VHSV.824 | FiA03.02 | 2002 | Baltic Sea/Finland | *Oncorhynchus mykiss* | 1-Id |
| AM086366 | FP.VHSV.825 | FiA04.02 | 2002 | Baltic Sea/Finland | *Oncorhynchus mykiss* | 1-Id |
| AM086367 | FP.VHSV.826 | FiA06.02 | 2002 | Baltic Sea/Finland | *Oncorhynchus mykiss* | 8-Id |
| AM086368 | FP.VHSV.827 | FiA07.02 | 2002 | Baltic Sea/Finland | *Oncorhynchus mykiss* | 1-Id |
| AM086369 | FP.VHSV.828 | FiA08.02 | 2002 | Baltic Sea/Finland | *Oncorhynchus mykiss* | 9-Id |
| AM086370 | FP.VHSV.829 | FiA09.02 | 2002 | Baltic Sea/Finland | *Oncorhynchus mykiss* | 1-Id |
| AM086371 | FP.VHSV.830 | FiA10.02 | 2002 | Baltic Sea/Finland | *Oncorhynchus mykiss* | 10-Id |
| AM086372 | FP.VHSV.831 | FiA11.02 | 2002 | Baltic Sea/Finland | *Oncorhynchus mykiss* | 11-Id |
| AM086373 | FP.VHSV.832 | FiA12.02 | 2002 | Baltic Sea/Finland | *Oncorhynchus mykiss* | 9-Id |
| AM086374 | FP.VHSV.833 | FiA13.02 | 2002 | Baltic Sea/Finland | *Oncorhynchus mykiss* | 11-Id |
| AM086375 | FP.VHSV.834 | FiA14.02 | 2002 | Baltic Sea/Finland | *Oncorhynchus mykiss* | 11-Id |
| AM086376 | FP.VHSV.835 | FiA15.02 | 2002 | Baltic Sea/Finland | *Oncorhynchus mykiss* | 1-Id |
| AM086377 | FP.VHSV.836 | FiA16.02 | 2002 | Baltic Sea/Finland | *Oncorhynchus mykiss* | 12-Id |
| AM086378 | FP.VHSV.837 | FiA17.02 | 2002 | Baltic Sea/Finland | *Oncorhynchus mykiss* | 13-Id |
| AM086379 | FP.VHSV.838 | FiA03.03 | 2003 | Baltic Sea/Finland | *Oncorhynchus mykiss* | 1-Id |
| AM086380 | FP.VHSV.839 | FiA18.03 | 2003 | Baltic Sea/Finland | *Oncorhynchus mykiss* | 14-Id |
| AM086381 | FP.VHSV.840 | FiU01.03 | 2003 | Baltic Sea/Finland | *Oncorhynchus mykiss* | 1-Id |
| AM086382 | FP.VHSV.841 | FiA03.04 | 2004 | Baltic Sea/Finland | *Oncorhynchus mykiss* | 1-Id |
| AM086383 | FP.VHSV.842 | FiA19.04 | 2004 | Baltic Sea/Finland | *Oncorhynchus mykiss* | 15-Id |
| AY546619 | FP.VHSV.234 | GE-1.2 | 1981 | Georgia | *Oncorhynchus mykiss* | 1-Ie |
| LN877207 | FP.VHSV.235 | TR-WS13G | 2005 | Turkey | *Scophthalmus maximus* | 2-Ie;  3-Ie |
| KM972680 | FP.VHSV.235 | TR-WS13G | 2005 | Turkey | *Scophthalmus maximus* | 4-Ie |
| KM972678 | FP.VHSV.1122 | Bolu/06 | 2006 | Turkey | *Oncorhynchus mykiss* | 5-Ie |
| KM972679 | FP.VHSV.492 | ckc-4 | 2009 | Turkey | *Scophthalmus maximus* | 6-Ie |
| LN877208 | FP.VHSV.1125 | NL59670 | 1987 | Netherlands | *Oncorhynchus mykiss* | 10-Ic |
| LN877209 | FP.VHSV.1126 | NL137609 | 1992 | Netherlands | *Oncorhynchus mykiss* | 364-Ia-7 |
| LN877210 | FP.VHSV.1127 | NL11008619-4 | 2011 | Netherlands | *Oncorhynchus mykiss* | 244-Ia-9 |
| JN180851 | FP.VHSV.1123 | UK-J167 | 2006 | United Kingdom | *Oncorhynchus mykiss* | 386-Ia-5 |
| LN877211 | FP.VHSV.1055 | V01-15CZ | 2015 | Czech Republic | *Oncorhynchus mykiss* | 389-Ia-8 |
| U28747 | FP.VHSV.328 | makah | 1988 | West coast of North America | *Oncorhynchus kisutch** | IVa |
