# Supplementary material for: Phylogeny of the Viral Hemorrhagic Septicemia Virus in European Aquaculture
Source: PLoS One. 2016 Oct 19;11(10):e0164475. doi: 10.1371/journal.pone.0164475 (PMC5070809; doi:10.1371/journal.pone.0164475)
Supplement: S2 Table — (DOCX) [file pone.0164475.s003.docx]

**S2 Table. Primer sequences.**

| **Name** | **Orientation** | **Nucleotide position** | **Sequence** (5ʹ🡪 3ʹ) |
| --- | --- | --- | --- |
|  |  | (*reference sequence* Y18263) |  |
| V2782f | forward | 2782–2801 | 5ʹ-ACT ACT ACA ATC GTG CCG TC-3ʹ |
| V3138f | forward | 3138–3157 | 5ʹ-TCA ACT CAG GTG TCC TCA TG-3ʹ |
| V3676f | forward | 3676–3693 | 5ʹ-GCA GGC CAT CAT CCC TGG-3ʹ |
| V4664r | reverse | 4664–4645 | 5ʹ-GTC ACA GTT GAG GTA GTT GC-3ʹ |
| V4211r | reverse | 4211–4192 | 5ʹ-CCA CTG TCA TAG ACA CTC TG-3ʹ |
| V3720r | reverse | 3720–3701 | 5ʹ-TGT CAC TGT GCA TGC CAT TG-3ʹ |
| V3280r | reverse | 3280–3261 | 5ʹ-CTC GAT AAG TCA CTC TGT GC-3ʹ |
